# Changes in symptom pattern in Meniere's disease by duration: the need for comprehensive management

**DOI:** 10.3389/fneur.2024.1496384

**Published:** 2024-11-08

**Authors:** Ilmari Pyykkö, Jing Zou, Nora Vetkas

**Affiliations:** ^1^Hearing and Balance Research Unit, Field of Otolaryngology, School of Medicine, Faculty of Medicine and Health Technology, Tampere University, Tampere, Finland; ^2^Department of Otolaryngology-Head and Neck Surgery, Center for Otolaryngology-Head and Neck Surgery of Chinese People's Liberation Army (PLA), Changhai Hospital, Second Military Medical University, Shanghai, China; ^3^Department of Gastroenterology, University Hospital of Helsinki, Helsinki, Finland

**Keywords:** episodic vertigo, quality of life, vestibular drop attacks, bilateral disease, balance, cognitive impairment

## Abstract

**Purpose:**

This retrospective study aimed to analyze the symptom profile of Meniere's disease (MD) patients, particularly focusing on the cessation of episodic vertigo and the disease's longitudinal course and the impact of major symptoms on quality of life (QoL).

**Methods:**

The study employed a cross-sectional design and was conducted on 365 out of 560 individuals with definite MD from the Finnish Vestibular and Meniere Federation, utilizing an internet-based questionnaire. Participants were surveyed on vertigo attacks, vestibular drop attacks (VDA), balance issues, selective cognitive complaints, hearing loss, and their effects on overall quality of life (QoL). The study population comprised 79.5% females and 20.5% males, with a mean age of 63 years and an average disease duration of 15.2 years.

**Results:**

The onset of MD was characterized by simultaneous hearing loss, vertigo, and tinnitus in 38% of participants. There was a significant delay in diagnosis for many, with 20% experiencing a delay of over 5 years. The frequency and duration of vertigo attacks generally decreased over time, with attacks becoming shorter and less severe as the disease progressed. Spontaneous remission from episodic vertigo occurred in 34% of participants variably throughout the course of MD. Of the participants 65.5% reported balance issues, and 34% experienced mild VDAs, with severe falls occurring in 10%. VDAs were more common with longer disease duration. Bilateral hearing loss developed in 34.5% of participants over the long term, with a higher risk associated with younger onset age, migraines, and family history of MD. Fatigue, anxiety, and depression were prevalent, particularly among younger participants. Cognitive impairments were linked to the severity of these symptoms and the presence of constant dizziness. QoL was significantly lower among participants with constant dizziness, with factors like fatigue, depression, VDA, and hearing loss contributing to this reduction.

**Conclusions:**

The study highlights the complexity of MD. While vertigo may spontaneously remit, other symptoms such as VDAs, balance issues, cognitive complaints, and hearing loss often persist and worsen over time. Assessing MD solely on primary symptoms like vertigo and hearing loss is insufficient; a comprehensive evaluation is necessary for effective management.

## Introduction

Meniere's disease (MD) is a disorder characterized by episodic vertigo, fluctuating hearing loss, tinnitus, and aural fullness. MD is thought to originate in the inner ear, as endolymphatic hydrops has been observed in histological preparations and through MRI imaging (1, 2). Although the etiology of MD is not fully understood, it is believed to arise from multiple etiological factors (3). Recent studies have associated MD with specific cytokine profiles and aberrant immunological genetic markers, suggesting an inflammatory etiology for the disease (4–7).

MD is a chronic condition with a prevalence in the general population ranging from 0.27 to 0.5% (8, 9), and there is currently no specific curative therapy available (2). The disease course is often unpredictable and is associated with severe-to-profound hearing loss, vertigo, dizziness, vestibular drop attacks (VDA), and balance and gait disturbances. Vestibular dysfunction, though less frequently observed, can also lead to cognitive impairments, including deficits in visuospatial abilities, processing speed, short-term memory, and executive function, resulting in a heterogeneous symptom profile (10, 11). Patients with MD frequently experience functional limitations and restrictions (12, 13), which contribute to a significant reduction in health-related quality of life (QoL) (14).

There have been few reports of spontaneous remission of vertigo attacks or periods of exacerbation in MD. Stahle et al. (15) observed that the frequency of vertigo episodes diminishes in the advanced stages of the disease. Similarly, Green et al. (16) suggested that the disease reaches a plateau between disease onset and 9 years of follow-up. Perez-Garrigues et al. (17) demonstrated that vertigo attacks tend to decrease annually, with 70% of patients becoming free of episodic vertigo in subsequent years. Silverstein et al. (18) evaluated 50 patients who declined surgical therapy for MD and found that 28 had complete control of vertigo within 2 years, and 35 achieved complete control after an average of 8.3 years. In Watanabe's study (19), the majority of 29 patients, including those with bilateral disease, experienced complete control of vertiginous spells after ten or more years of follow-up, though the long-term outcome of hearing preservation was not well-maintained. In a study by Tokumasu et al. (20), only 29% of MD patients were free from vertigo attacks after 7 years of follow-up.

Both clinical data and imaging evidence suggest that MD exists on a continuum, ranging from monosymptomatic cases to full-blown bilateral involvement. Interestingly, even in unilateral MD cases, up to 70% of patients may exhibit bilateral endolymphatic hydrops on MRI (21–23). Paparella and Griebie (24) analyzed the audiometric configurations of a randomly selected group of 360 patients with unilateral MD and found that in approximately half of those with bilateral disease, the second ear became involved within 2 years of the first ear's involvement. In another 27%, the second ear became involved after a period of 5 years or more, with the overall incidence of bilateral MD estimated at 32%. While sensorineural hearing loss is suspected to be an early indicator of second ear involvement, the bilaterality of MD varies significantly depending on the nature of symptoms and the diagnostic criteria employed (25).

The objective of this retrospective study was to examine the symptom profile in MD patients, with a particular focus on the cessation of episodic vertigo and the longitudinal course of the disease.

## Materials and methods

### Study design

A cross-sectional study design was employed to analyze registry data obtained from the Finnish Vestibular and Meniere Federation (VMF). This registry comprises detailed data collected by the VMF from its members during the establishment of a computer-based diagnostic and peer support program between 1998 and 2016 (26, 27) (Appendix, questionnaire 1). According to Finnish law, anonymous registry data collected by patient associations does not require ethical approval. However, the researchers secured permission from the VMF to analyze the data for the purposes of this study.

### Participants

The original VMF database included data from 1,050 members. To evaluate the diagnostic accuracy of MD within the group, the members' diagnoses were assessed using an expert program and compared with the diagnostic criteria established by the American Academy of Otolaryngology–Head and Neck Surgery (AAO-HNS, 1995). The evaluation indicated that 97% of the members had definite MD, while 2.7% had probable MD (26, 27). The patient's medical history was entered into the inference engine to evaluate the accuracy of the diagnosis. The program operates using two categories of information: necessary and supportive data. The classification of signs depends on the specific disease; thus, one sign may be categorized as necessary, while another may be considered supportive. A necessary sign indicates that, if present, the disease is being actively considered as a possible diagnosis, such as Menière's disease. Supportive signs suggest the condition but are not required for the diagnosis. The program employs a pattern recognition algorithm, detailed in previous studies (26, 27). Based on the inference engine's analysis, different probability scores are generated. One score reflects the outcome when all necessary data have been provided, while the other accounts for missing data, leading to uncertainty. To calculate these probability values, the patient's data were compared with an optimal dataset.

To gather additional data for the present study, an electronic questionnaire was designed and distributed via email to the 1,050 VMF members (Appendix, questionnaire 2). Four reminders were sent to those who did not initially respond. A total of 560 members completed the survey. To further assess the effects of medical therapy, the disease course, and the impact of MD, a follow-up survey was conducted with a new questionnaire, to which 365 members (65.1% of the original respondents) replied (Appendix, questionnaire 3). The VMF provided the data anonymously to the researchers for further analysis. Additionally, the VMF's ethical committee approved the use of this new registry data (Protocol No: 2022-06-08). Audiograms were available for 156 participants from the original database. Of the study participants, 290 (79.5%) were female, and 75 (20.5%) were male, reflecting the gender distribution of the VMF. The mean age was 63 years (range 13–91 years), and the mean duration of the disease was 15.2 years (range 0.5–48 years).

### Data collection

The survey questions focused on the impact of MD, socioeconomic aspects, and specific complaints related to the course and nature of vertigo attacks in MD. Vertigo was assessed by the date of the last attack, the frequency and duration of the attacks, and the impact of these attacks. Balance problems were queried based on their presence in the last 2 years, their characteristics, and their impact. The impact of postural complaints was rated on a five-point scale ranging from “no impact” to “severe impact,” defined by interference with activities based on the 15D instrument (28). VDA was defined as brief episodes of sudden postural instability unrelated to head movements. VDAs were classified as mild when patients experienced a sudden feeling of instability, moderate when balance issues were associated with a near-fall but were prevented by seeking support, and severe when the patient fell to the ground (29, 30). General health-related QoL was measured using the Visual Analog Scale (VAS) from the EuroQol EQ-5D-3L questionnaire (31). The impact of nine major problems associated with MD was also assessed and rated. Furthermore, participation in physiotherapy and psychotherapy was recorded, along with assessments of vitality, depression, and anxiety using the 15D instrument (28).

Medical therapy was examined by asking participants about the types of medications or therapies used, including their dosages. Participants could report information about up to 10 different drugs. The survey also included questions regarding the type of intratympanic therapy (e.g., corticosteroids, gentamicin) used (Table 1). Additionally, questions were posed about vestibular and visual training, including the types, frequency, and whether the training was guided or self-administered.

**Table 1:** Anxiety, depression and fatigue rating and number of subjects in each impact class.

**Grade**	**Depression**	**Anxiety**	**Fatigue**
No	121 (33%)	133 (36%)	64 (18%)
Slightly	175 (48%)	164 (45%)	179 (49%)
Moderately	53 (15%)	52 (14%)	85 (23%)
Severely	9 (3%)	11 (3%)	27 (7%)
Very severely	7 (2%)	5 (1%)	10 (3%)
All	365 (100%)	365 (100%)	365 (100%)
*Age of subject* ANOVA	*F* = 5.31, *p* < 0.001	*F* = 8.76, *p* < 0.001	*F* = 4.57, *p* = 0.001
*Duration of MD* Kruskal-Wallis test	*H* = 13.78, *p* = 0.032	*H* = 19.2, *p* = 0.004	*H* = 13.07, *p* = 0.042
*Vertigo/No vertigo* Kruskal-Wallis test	*H* = 9.84, *p* = 0.002	*H* = 4.69, *p* = 0.030	*H* = 7.33, *p* = 0.007

Effect of age (ANOVA) and duration of Meniere's disease (Kruskal-Wallis test) test outcome between the impact class groups.

### Statistical analysis

Data on the diagnosis of unilateral or bilateral MD were based on self-reported hearing loss, and the number of participants with unilateral or bilateral disease was further examined using existing audiograms. Missing data were analyzed, and where necessary, imputation was performed. Logistic regression was used to examine factors influencing the complaint profiles in MD for binary variables, while linear regression analysis was applied for continuous variables. The Kruskal-Wallis *H*-test was employed to examine differences between complaint groups and therapy groups. Comparisons between groups for categorical variables were made using the Mann-Whitney *U*-test, and for continuous variables using Student's *t*-test.

## Results

### Age of onset of MD, duration of MD, and last vertigo attack in MD

In 38% of the subjects, MD onset was characterized by the simultaneous appearance of hearing loss, vertigo, and tinnitus. The temporal gap between the onset of vertigo and hearing loss was prolonged in many cases, irrespective of whether the initial symptom was vertigo or hearing loss. In 17% of the participants, a diagnosis of definitive MD was established within the first year of symptom onset. For 21% of the participants, the diagnostic delay for definitive MD ranged from 1 to 4 years. In 11% of the participants, the time lapse between the onset of hearing loss and vertigo was 5–10 years, while in 9%, the delay exceeded 10 years. Consequently, in ~20% of the participants, the time delay between symptom onset and the definitive diagnosis of MD exceeded 5 years (Figure A1). The mean age of MD onset was 47.8 years (range 10–79 years).

**Figure 1:**
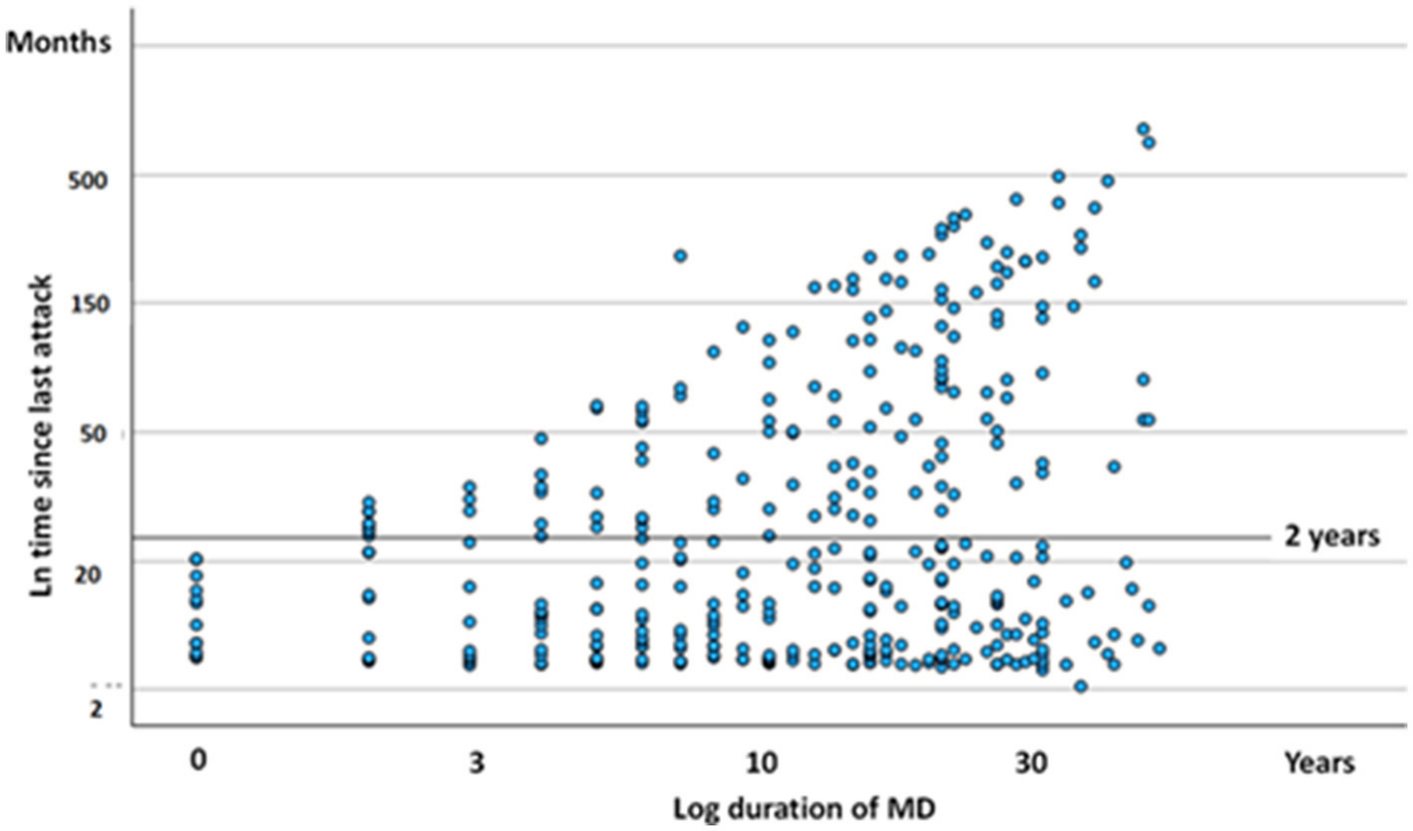
Scatter plot diagram of duration since last attack of episodic vertigo (ordinate) expressed against course of MD. Each dot represent one subject. The scales are logarithmic.

Altogether, 123 out of 365 participants (34%) were free of vertigo attacks for more than 2 years at the time of the study. Figure 1 illustrates the distribution of attack-free periods in relation to the duration of MD. Analysis of variance revealed no significant difference in the cessation of episodic vertigo based on the duration of MD (*F* = 1.229, df = 6, *p* = 0.262). The clustering of dots near the bottom of the *Y*-axis (near zero) indicates that several participants experienced a recent vertigo attack, regardless of the disease's progression stage.

When evaluating the influence of age (*t* = 3.42, *p* = 0.006) and the duration of MD (*t* = 2.56, *p* = 0.005) on the cessation of episodic vertigo, both variables were found to be significantly correlated with the pausing of vertigo episodes. This suggests that older individuals and those with a longer disease duration were more likely to experience periods without episodic vertigo. However, the age at which individuals initially developed MD did not significantly influence the pausing of episodic vertigo (*t* = 0.19, *p* = 0.363).

### Character of dizziness and vertigo in MD

Participants reported experiencing both episodic vertigo and constant dizziness, with or without accompanying vertigo episodes. Specifically, 26.8% of participants reported no vertigo spells or dizziness in the past 2 years, while 3% experienced constant dizziness without episodic vertigo. Additionally, 21.6% had both episodic vertigo and constant dizziness, and 48% experienced episodic vertigo without constant dizziness (Figure A2).

### Duration of the vertigo attacks

**Figure 2:**
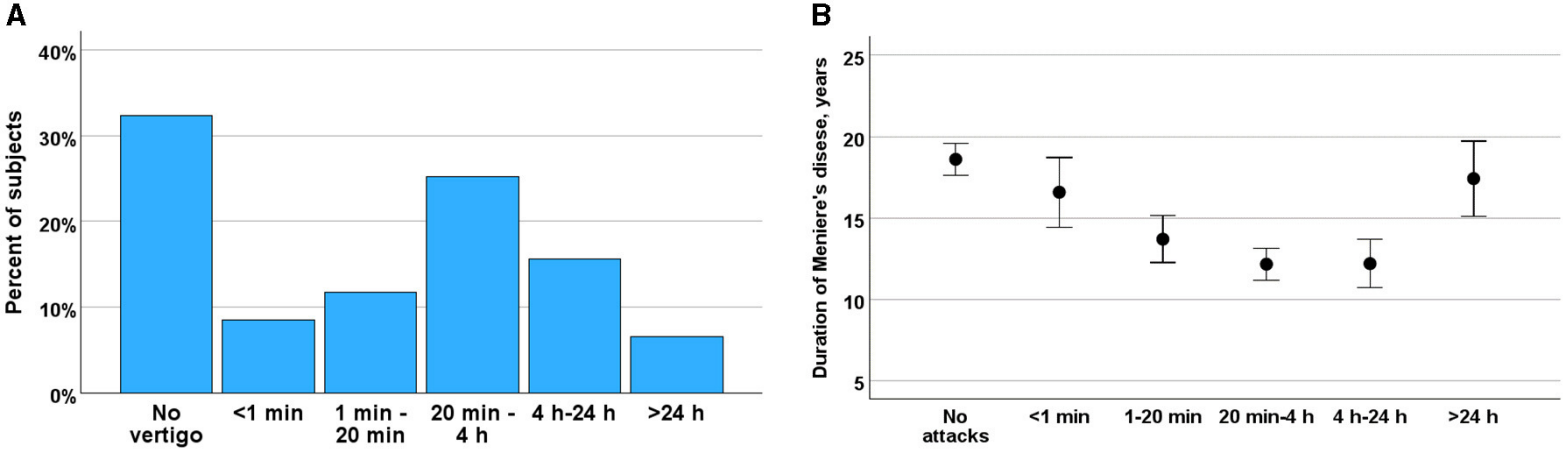
**(A)** Duration vertigo attacks among participants rated from attacks <1 min to >24 h in percent of the subjects. **(B)** Changes in frequency of vertigo during course of MD (ordinate) and by frequency of vertigo attacks (abscissa). Means and SE are given.

The duration of vertigo episodes in MD was found to be highly variable (Figure 2A). Notably, only 40.7% of vertigo attacks conformed to the current diagnostic criteria for MD (32), suggesting that the characteristics of vertigo in MD may change over time (*r* = −0.197, *p* < 0.001). Vertigo episodes lasting between 20 minu and 4 h were more prevalent in the early stages of the disease, whereas brief episodes of ~1 min were more commonly observed in the later stages (Figure 2B). Additionally, prolonged vertigo attacks were more frequently reported in advanced stages of the disease (ANOVA, *F* = 5.461, *p* < 0.001). The brief vertigo attacks were associated with change in position of head and resulted in weak (Kruskal-Wallis test, *H* = 4.30, *p* = 0.038) or moderate vertigo (*H* = 5.47, *p* = 0.019) as in BPPV. This association was not met with strong vertigo (*H* = 0.38, *p* = 0.845).

### Frequency of the vertigo attacks

Episodic vertigo attacks in MD were most frequently reported to occur less than once per month (35.6%), followed by less than once per year (13.7%) and monthly (10.4%). Vertigo attacks occurring weekly (4.1%) and daily (1.4%) were relatively rare (Figure A3). An analysis of the trends in vertigo frequency throughout the course of MD revealed a general decrease in attack frequency over time (ANOVA *F* = 4.4, *p* < 0.001). However, no specific frequency of vertigo attacks was significantly associated with the duration of MD (Bonferroni test, *p* = n.s. between different attack frequencies).

### Impact of the vertigo attack

**Figure 3:**
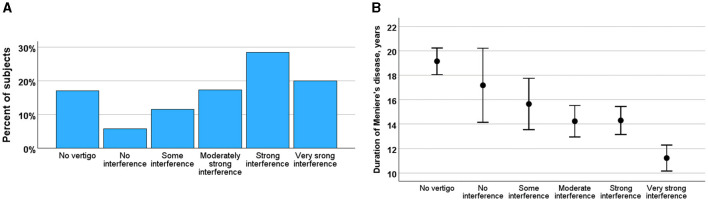
**(A)** Impact of vertigo rated by interference of daily activities (from no interference to very strong interference) in percent of the subjects. **(B)** Changes of impact of vertigo during course of MD (ordinate) by impact of vertigo rated by interference of daily activities (abscissa). Means and SE are given.

The majority of participants rated vertigo as significantly strong, with 64% reporting that the attacks interfered with their daily activities. Specifically, 39% of the participants needed to rest during an attack, and 25% experienced difficulties even while resting (Figure 3A). In contrast, 6% of the participants reported no interference with daily activities, and 12% reported only mild interference. The severity of the attacks decreased over the course of MD, as indicated by a negative correlation between the course of the disease and the impact of attacks (*r* = −0.141, *p* < 0.001) (Figure 3B).

### Vestibular drop attacks in MD

Among the participants, 172 (47%) did not report any VDA during the last 2 years. However, 123 participants (34%) experienced brief, mild postural perturbations, unassociated with head movement and without the risk of falling. A Tumarkin-like fall to the ground occurred in 37 participants (10%), while another 37 participants (10%) were able to prevent a fall by seeking support (Figure A4A). Although VDA occurrences were infrequent and predominantly occasional (35%), they could result in severe consequences for some participants (Figure A4B). Among those who fell to the ground, 24 reported syncope, with 3 cases being eye-witnessed. No significant association was observed between the prevalence of syncope and the duration of MD (ANOVA, *F* = 1.15, *p* = 0.307).

### Balance problems in MD

**Figure 4:**
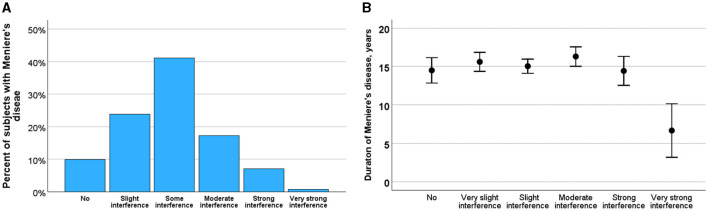
**(A)** Severity of balance problems rated as no, slight, some, moderate, severe and very severe impact on daily life. **(B)** Effect of course of Meniere's disease on the impact of balance problems. Means and SE are shown.

Over the past 2 years, 238 participants (65.5%) reported experiencing balance problems outside of vertigo attacks. These balance issues were characterized into different types: 23% described them as a slow swaying sensation, and 7% as a rocking sensation, both consistent with Mal de Debarquement Syndrome (MdDS). Additionally, 49% of participants reported experiencing balance problems akin to tripping. Figure 4A illustrates the varying degrees to which these balance problems impacted daily life. Most participants reported very slight (24%) or slight (42%) difficulties in carrying out daily activities. Meanwhile, 18% experienced moderate restrictions, and 8% reported severe or very severe limitations in their daily activities.

Figure 4B illustrates the relationship between balance problems and the progression of MD, indicating how the impact may evolve as the disease advances. ANOVA analysis revealed that the impact of balance problems did not significantly change with disease progression (*F* = 0.585, *p* = 0.711) or with increasing age (*F* = 1.150, *p* = 0.333). However, the impact of balance problems was significantly correlated with the duration of attack-free periods (Kruskal-Wallis test, *H* = 14.4, *p* = 0.013).

### Longitudinal course of hearing loss

**Figure 5:**
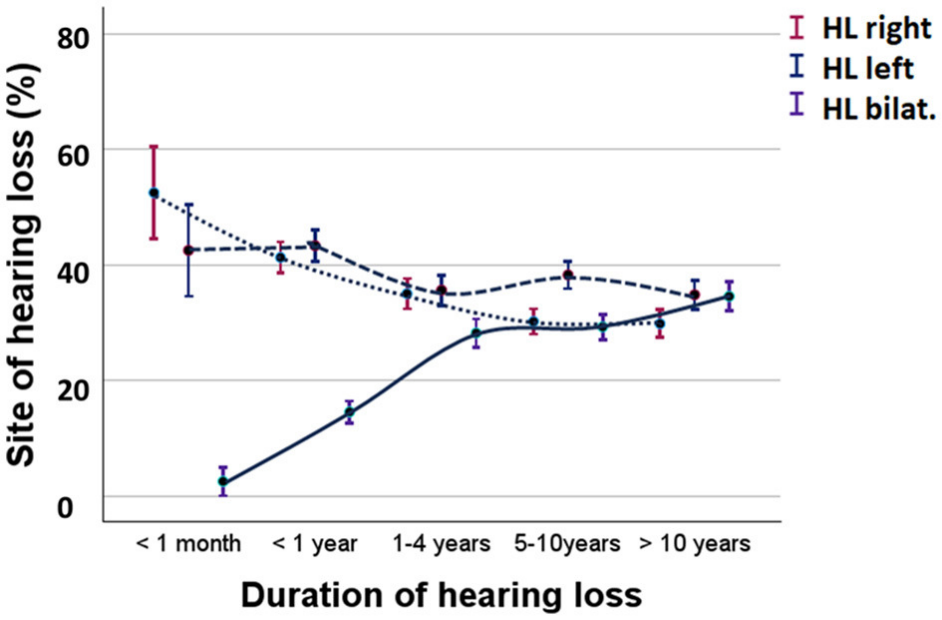
Percent of participants with unilateral or bilateral hearing loss during MD. Solid lines represent bilateral hearing loss. Broken lines represent unilateral hearing loss. HL right, hearing loss in the right ear; HL left, hearing loss in the left ear; HL bilat, bilateral hearing loss. Means and standard error are shown.

Figure 5 compares the percentages of participants experiencing unilateral vs. bilateral hearing loss due to MD. Initially, 2.4% of participants had bilateral hearing loss, which increased to 13.8% within the first year, 27.7% within 4 years, 28.7% after 5–10 years, and 34.5% after 10 years (Kruskal-Wallis test, *H* = 14.9, *p* < 0.001).

In a selected group of participants (*n* = 156), audiograms were available, allowing for an analysis of the development of hearing loss in patients with MD. The mean hearing levels at frequencies of 500, 1,000, and 2,000–4,000 Hz were used for the analysis. In cases of bilateral MD, the hearing level represents the average of both ears. Patients with a history of bilateral MD longer than 10 years had an average hearing loss of 51.2 dB (SE = 2.9 dB), while those with unilateral hearing loss showed an average of 49.3 dB (SE = 9.6 dB) in the right ear and 54.0 dB (SE = 5.9 dB) in the left ear. The measured hearing loss corresponded with the observed prevalence of experienced hearing loss (Figure 5). Cessation of vertigo did not significantly influence hearing loss (Student's *t*-test, *t* = 1.251, *p* = 0.080). Logistic regression analysis explored the risk factors for bilateral hearing loss, revealing that migraine (*p* = 0.09), onset of MD at a younger age (*p* < 0.001), and having siblings with MD (*p* = 0.003) were associated with an increased risk of bilateral hearing loss.

### Anxiety, depression, and fatigue during the longitudinal course of MD

Table 1 presents data on the distribution of depression, anxiety, and fatigue across varying severity levels, along with statistical analyses (ANOVA and Kruskal-Wallis tests) exploring how these symptoms relate to age, duration of MD, and the presence of vertigo. Depression, anxiety, and fatigue were reported by 67, 64, and 82% of subjects with MD, respectively (Table 1), with slight symptoms being the most common for all three conditions. These complaints were inversely related to age, with older participants experiencing these symptoms less severely than younger participants. The severity of depression, anxiety, and fatigue was significantly related to age, duration of MD, and the presence of vertigo.

In a logistic regression analysis that considered various MD-related complaints, VDA were the only factor significantly associated with the use of antidepressants (*p* = 0.007), with an odds ratio of 3.2.

### Health-related quality of life and rating of the impact on different complaints in MD

Participants who had been attack-free for 2 years or more reported a QoL score of 69.6, while those experiencing episodic vertigo reported a QoL score of 68.3. There was no significant difference in QoL between those with episodic vertigo and those without. However, when comparing these groups to participants with constant dizziness, significant differences in QoL were observed (ANOVA, *F* = 4.79, *p* = 0.006). The Bonferroni test revealed that participants with episodic vertigo and constant dizziness, as well as those with constant dizziness alone, had significantly lower QoL compared to those without vertigo (*p* = 0.014) and those with vertigo alone (*p* = 0.019). This indicates that constant dizziness, with or without vertigo episodes, significantly reduces QoL (Figure A5).

The duration of MD (*r* = 0.252, *p* < 0.001) and age (*r* = −0.234, *p* < 0.001) were both associated with reduced quality of life (QoL), indicating that older individuals or those with a longer history of MD had lower QoL scores. In contrast, the duration of episodic attack-free periods did not have a significant impact on QoL (ANOVA, *F* = 1.007, *p* = 0.421).

In a linear regression analysis, fatigue, depression, VDA, hearing loss, and working with display instruments (such as personal computers or television) were identified as significant factors in the model (*p* < 0.001) that contributed to a reduction in QoL. These variables collectively explained 24.1% of the variability in QoL (*r* = 0.491) (Table 2).

**Table 2:** Impact of cognitive symptoms and Meniere's disease symptoms in reduction of health-related quality of life in linear regression analysis.

**Complaint**	**Beta**	***t***	**Significance**
Constant	89.07	35.3	<0.001
Fatigue	−6.51	−4.09	<0.001
Depression	−4.71	−2.82	0.005
Severity of VDA	−2.87	−2.40	0.017
Hearing loss	−5.51	−2.33	0.020
PC and TV usage problems	−5.59	−2.10	0.036

**Figure 6:**
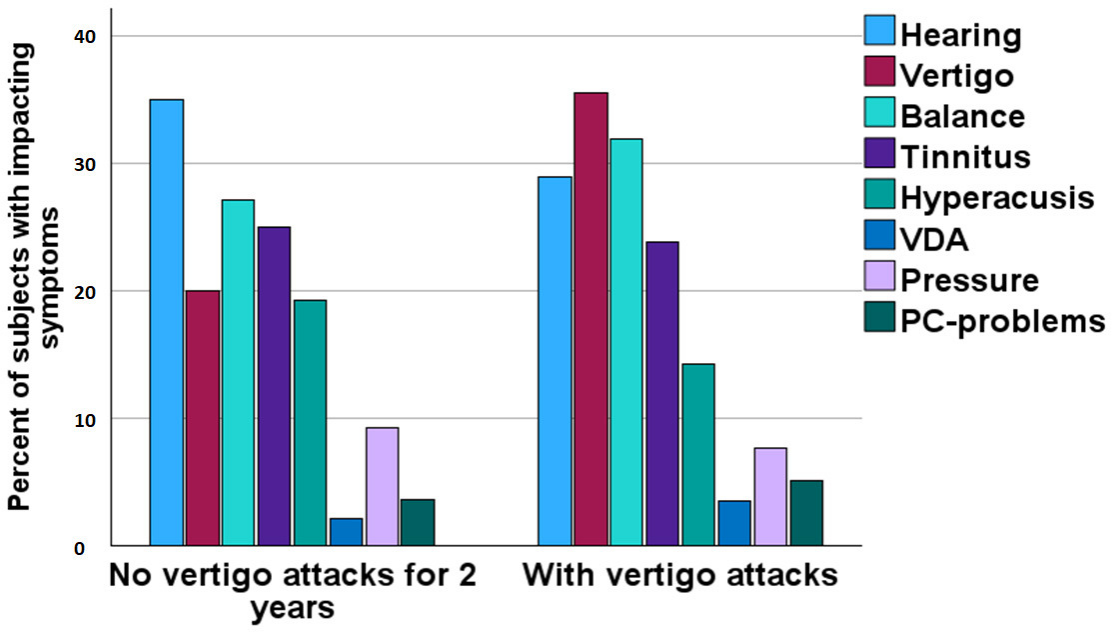
Rating of most severe complaints among participants divided on absence of episodic vertigo for 2 years or more.

Participants were asked to rate the impact of the cardinal symptoms of MD from most severe to least problematic on a scale of 1–9. If a participant did not experience a particular symptom, they could leave the rating blank. Figure 6 illustrates the impact of these symptoms on the lives of participants with episodic vertigo compared to those who had been free of episodic vertigo for at least 2 years. A lower score indicates a lesser impact on the participant's life. The analysis showed that the cessation of episodic vertigo did not result in significant differences in the impact of the various symptoms of MD (Mann-Whitney *U*-test, no significant differences).

## Discussion

We conducted a cross-sectional study involving 365 out of 560 participants from a patient organization to evaluate the long-term course of MD. The study revealed that, in the long term, 34.5% of participants developed bilateral hearing loss. Episodic vertigo abated in 34% of participants for more than 2 years, with the cessation of episodic vertigo occurring at varying time points during the disease. Over time, the nature of vertigo attacks changed, with shorter attacks becoming more common, and the frequency of attacks generally decreasing. The severity of vertigo also diminished in the long run. Neither very short nor very long attacks have been reported in prior studies (17). Our findings also indicated that the impact of vertigo attacks became milder as the duration of MD increased, consistent with previous literature. The changing frequency and duration of vertigo episodes with disease progression provide valuable insights into symptom evolution and highlight the need for adaptable management strategies as MD advances and the brief vertigo attacks were associated with change in position of head as in BPPV. This finding is important since patients with MD commonly develop BPPV in the final stages and we observed.

While the frequency of VDA was associated with longer duration of MD, the overall frequency of VDA did not change significantly during the disease. VDAs were mild in 34% of cases, moderate in 10%, and severe enough to cause falls in 10%. Postural problems were associated with the cessation of vertigo. Changes in QoL were not related to remission from episodic vertigo but were instead explained by cognitive consequences of MD, such as fatigue, depression, visual problems, VDA, and hearing loss.

The results indicate that the impairment caused by MD is complex and often misunderstood, even among professionals. Therefore, MD should not be considered “cured” when “spontaneous remission” of vertigo occurs. As descriptions of the quality of vertigo and dizziness described by the patients are based on personal experience, they may not match the doctor's experience of complaint classifications, especially in classifying patients with dizziness. The natural course of MD introduces problems related to mood, cognition, participation restrictions, and activity limitations, all of which impact disability and QoL. In this study, MD began with the classic symptoms in only 40% of patients, confirming previous reports of challenges in diagnosing the disease in monosymptomatic cases (22, 23).

In the present study, the bilaterality of hearing loss was ~14% after 1 year, increasing to 34% over the long course of MD, which is consistent with previous reports (33). Consistent with our results, previous studies have associated the risk of developing bilateral MD with younger age of onset, the presence of migraines, familial history of MD, history of ear infections, autoimmune diseases, and genetic variants (34, 35). Shojaku et al. (20) reported bilateral MD in 9% of a series of 958 patients with definite MD. Kodama et al. (36) studied 480 cases of MD, finding that 135 (28%) showed fluctuating cochlear symptoms in the contralateral ear, classifying these cases as bilateral involvement. The present study revealed that unilateral hearing loss tended to progress to bilateral hearing loss in 1–4 years, which is shorter than previous reports (15, 25, 37). This topic still requires further investigation.

Eraslan Boz et al. (38) employed a comprehensive array of neuropsychological measures and found that cognitive deficits in MD were associated with impairments in attention, visual memory recall, visuospatial construction, and planning skills, among other cognitive domains. The unpredictability of MD attacks, along with chronic dizziness and ear symptoms, may exacerbate mental stress, contributing to conditions such as anxiety, depression, panic disorder, and dyssomnia (39). These factors may mediate cognitive impairment (11, 40).

In the present study, we focused on vestibular-associated cognitive impairments, including mood, attitude, balance, and visual disturbances. Fatigue was the most commonly reported problem, with 38% of participants experiencing moderate to very severe fatigue. Additionally, moderate to very severe symptoms of depression and anxiety were reported by 20 and 18% of participants, respectively. These complaints were age-related, with older individuals experiencing these symptoms less severely than younger patients. The duration of episodic vertigo-free periods also differed with respect to the severity of these complaints. Notably, participants with longer vertigo-free periods more frequently used antidepressant or anxiolytic medications, suggesting that vertigo was not the primary reason for medication. The ability to cope with MD may explain the individual variability in mental stress-related complaints. A previous study found that cognitive improvements correlated with decreases in dizziness handicap index scores (39).

Balance problems were reported by 65.5% of participants, who experienced these issues outside of episodic vertigo over the past 2 years. These balance problems were characterized in different ways: 30% of participants described them as a slow swaying or rocking sensation, consistent with the diagnostic criteria for Mal de Debarquement Syndrome (MdDS) (41). We did not observe that the complaints of MdDS would be associated with any triggering incident and we consider the MdDS to be spontaneous type and caused by deranged otolith-semicircular canal interaction (41). These balance issues were inversely correlated with the duration of attack-free periods, indicating improvement in symptoms following the cessation of vertigo.

Participants who experienced both episodic vertigo and constant dizziness frequently reported visual problems, difficulties with computer use, and fatigue, suggesting impairments in cognitive function. Persistent Postural Perceptual Dizziness (PPPD) manifests with one or more symptoms of dizziness, unsteadiness, or non-spinning vertigo that are present on most days for at least 3 months or more and are exacerbated by upright posture, active or passive movement, and exposure to moving or complex visual stimuli (42). PPPD unifies key features of chronic subjective dizziness, phobic postural vertigo, visual subjective vertigo, and related disorders. The outcome of the present study indicates that constant dizziness and episodic vertigo are complaints of the similar kind of disorder and cognitive complaints in MD and PPPD may describe involvement of neural network in of vestibular disease process. So far, there is insufficient evidence to separate constant dizziness in MD from PPPD.

We hypothesize that constant dizziness is a sign of cognitive dysfunction closely associated with vestibular system malfunction (43). Therapeutic approaches for these cognitive complaints remain poorly understood, and further research in this area is needed (30). Developing effective therapies for cognitive complaints is crucial, as these factors are key determinants of quality of life in MD patients.

A recent consensus document in the USA advises against physiotherapy for periodic vertigo but supports the use of information dissemination, steroids, balance rehabilitation, and ablative treatment of vestibular function (44). Notably, the consensus documents, like other therapeutic regimens, do not recognize constant dizziness or cognitive problems as components of MD. Some efforts have been made to address these issues, such as the use of meditation (45). Adding cognitive-behavioral therapy to self-administered vestibular rehabilitation exercises in patients with phobic vertigo showed a positive short-term effect, although this effect was not sustained at 1-year follow-up (46). Other studies on patients with continuous subjective vertigo have shown more promising results (46). Regarding Persistent Postural-Perceptual Dizziness (PPPD), management has included effective communication and tailored treatment strategies, such as vestibular rehabilitation and cognitive-behavioral therapy (47, 48). For MdDS, a special therapy with visual vestibular interaction training has been suggested and with such therapy about 70% of the vestibular patients seem to respond positively (49). The implementation of a home-based virtual reality protocol for rehabilitation has also shown potential in improving balance control and QoL in the presence of cognitive decline (50).

The prevalence of VDA in patients with MD has been previously estimated to be ~13%, with these attacks often leading to falls (51). The pathophysiology of VDA is believed to involve sudden alterations in the function of the otolith organs, specifically the utricle or saccule. These changes lead to an incorrect perception of gravitational reference, causing reflex movements that misalign the body relative to Earth's true gravity, resulting in a sensation of being thrust to the ground. In the majority of MD patients, VDA occurs in a less severe form (29, 52). VDAs are also associated with gait problems and postural instability (30), although disequilibrium during attack-free periods or persistent disequilibrium is primarily observed in the later stages of the disease (53). Our study confirmed that the occurrence of VDAs is associated with a longer duration of MD.

In elderly patients, particularly when associated with syncope, VDAs may be misinterpreted by healthcare providers as part of a “geriatric syndrome” due to their multi-symptomatic presentation. A chart audit by Kwong and Pimlott (54) found that 46% of elderly patients presenting with vertigo during primary consultations were either improperly diagnosed or their symptoms were attributed to the natural aging process. Moreover, healthcare professionals often lack awareness of the association between inner ear disorders, particularly drop attacks with falls, postural instability, and syncope.

## Limitations of the study

We found that the terms “vertigo” and “dizziness” are often misinterpreted by patients, being conflated with balance and gait problems, cognitive complaints, and light-headedness. This misinterpretation has been reported in previous studies as well (55). To address this, we used specific questions to further characterize the type of vestibular complaint, differentiating between episodic vertigo (characterized by rotatory attacks with a spinning sensation) and dizziness. Defining the “attack-free period” is also challenging, as balance problems, dizziness, and VDA can fluctuate over time and mostly the patients experience better and worse days.

Given that the current study is based on self-reports, several limitations must be acknowledged. The questionnaire was anonymous, preventing us from posing additional focused questions to participants or retrieving retrospective hospital records. As a result, the findings of this study should be interpreted with caution and regarded as exploratory.

We also had limited access to audiometry data, with only 156 out of 365 subjects providing such information, which constrained our ability to include comprehensive audiometric data. Unlike most studies that report the worst hearing levels across multiple audiograms, our results are based on self-reported hearing loss in either one or both ears. This limitation may introduce bias, particularly in patients with fluctuating hearing loss, who might perceive their hearing as normal despite audiometric evidence of “progressive hearing loss” or in age related hearing loss.

Additionally, the study sample had a predominance of female participants, reflecting the membership of the patient organization of VMF, where females are more socially active in health-related matters. However, there appears to be no significant difference in the complaint profile based on gender, suggesting that this gender imbalance may not have biased the study's outcomes.

## Conclusion

The findings from this study provide valuable insights into the long-term course of Meniere's disease (MD) and its multifaceted impact on patients' lives. The study highlighted that while spontaneous remission of vertigo occurs in approximately one-third of MD patients, this does not equate to a resolution of the disease. Other symptoms, including vestibular drop attacks (VDAs), balance issues, cognitive impairments, and bilateral hearing loss, continue to evolve and affect patients' quality of life (QoL). These results underscore the need for a more comprehensive approach to the diagnosis and management of MD, one that considers the full spectrum of symptoms beyond vertigo.

The variability in symptom progression and the development of bilateral disease, as well as the cognitive and psychological impacts observed, suggest that MD is a complex and heterogeneous condition. This complexity can make diagnosis and treatment challenging, requiring personalized management strategies that address both the physical and psychological aspects of the disease.

Further research is needed to explore the underlying mechanisms driving the progression of MD, particularly the factors leading to bilateral involvement and the cognitive and psychological sequelae associated with the condition. Additionally, the development of therapeutic interventions that target the broader spectrum of MD symptoms, including constant dizziness, cognitive decline, and psychological distress, is crucial for improving patient outcomes.

## Data Availability

The raw data supporting the conclusions of this article will be made available by the authors, without undue reservation.

## References

[1] Zou J Pyykkö I Bretlau P Klason T Bjelke B. *In vivo* visualization of endolymphatic hydrops in guinea pigs: magnetic resonance imaging evaluation at 47 tesla. Ann Otol Rhinol Laryngol. (2003) 112:1059–65. 10.1177/000348940311201212 14703111

[2] Nakashima T Pyykkö I Arroll MA Casselbrant M Foster C Manzoor N. Meniere's disease. Nat Rev Dis Primers. (2016) 2:16028. 10.1038/nrdp.2016.28 27170253

[3] Frejo L Soto-Varela A Santos-Perez S Aran I Batuecas-Caletrio A Perez-Guillen V. Clinical subgroups in bilateral Meniere disease. Front Neurol. (2017) 8:528. 10.3389/fimmu.2017.01739 27822199 PMC5075646

[4] Zou J Zhao Z Zhang G Zhang Q Pyykkö I MEFV. IRF8, ADA, PEPD, and NBAS gene variants and elevated serum cytokines in a patient with unilateral sporadic Meniere's disease and vascular congestion over the endolymphatic sac. J Otol. (2022) 17:175–81. 10.1016/j.joto.2022.03.001 35847575 PMC9270563

[5] Zou J Zhang G Li H Zhao Z Zhang Q Pyykkö I. Multiple genetic variants involved in both autoimmunity and autoinflammation detected in Chinese patients with sporadic Meniere's disease: a preliminary study. Front Neurol. (2023) 14:1159658. 10.3389/fneur.2023.1159658 37273692 PMC10232973

[6] Frejo L Lopez-Escamez JA. Cytokines and inflammation in Meniere disease. Clin Exp Otorhinolaryngol. (2022) 15:49–59. 10.21053/ceo.2021.00920 35124944 PMC8901949

[7] Gallero-Martinez A Requena T Roman-Naranjo P Lopez-Escamez JA. Excess of rare missense variants in hearing loss genes in sporadic Meniere disease. Front Genet. (2019) 10:76. 10.3389/fgene.2019.00076 30828346 PMC6385525

[8] Havia M Kentala E Pyykkö I. Prevalence of Meniere's disease in general population of Southern Finland. Otolaryngol Head Neck Surg. (2005) 133:762–8. 10.1016/j.otohns.2005.06.015 16274806

[9] Tyrrell JS Whinney DJ Ukoumunne OC Fleming LE Osborne NJ. Prevalence, associated factors, and comorbid conditions for Meniere's disease. Ear Hear. (2014) 35:e162–9. 10.1097/AUD.0000000000000041 24732693

[10] Gürkov R Pyykkö I Zou J Kentala E. What is Menière's disease? – A contemporary re-evaluation of endolymphatic hydrops. J Neurol. (2016) 263(Suppl. 1):71–81. 10.1007/s00415-015-7930-1 27083887 PMC4833790

[11] Xie D Welgampola MS Miller LA Young AS D'Souza M Breen N. Subjective cognitive dysfunction in patients with dizziness and vertigo. Audiol Neurootol. (2022) 27:122–32. 10.1159/000518188 34518461

[12] Stephens D Pyykkö I Varpa K Kentala E. Self-reported effects of Menière's Disorder on the individual's life: a qualitative analysis. Otol Neurotol. (2010) 31:335–8. 10.1097/MAO.0b013e3181bc35ec 19841603

[13] Levo H Stephens D Poe D Kentala E Pyykkö I. Use of ICF in assessing the effects of Meniere's disorder on life. Ann Otol Rhinol Laryngol. (2010) 119:583–9. 10.1177/000348941011900903 21033024

[14] Pyykkö I Manchaiah V Zou J Levo H Kentala E. Impact evaluation and association with EuroQol 5D health-related utility values in Ménière's disease. Springerplus. (2015) 4:717. 10.1186/s40064-015-1527-0 26636005 PMC4656266

[15] Stahle J Friberg U Svedberg A. Long-term progression of Meniere's disease. Am J Otol. (1989) 10:170–3. 2750865

[16] Green JD Jr Blum DJ Harner SG. Longitudinal follow-up of patients with Menière's disease. Otolaryngol Head Neck Surg. (1991) 104:783–8. 10.1177/019459989110400603 1908968

[17] Perez-Garrigues H Lopez-Escamez JA Perez P Sanz R Orts M Marco J. Time course of episodes of definitive vertigo in Meniere's disease. Arch Otolaryngol Head Neck Surg. (2008) 134:1149–54. 10.1001/archotol.134.11.1149 19015442

[18] Silverstein H Smouha E Jones R. Natural history vs. surgery for Menière's disease. Otolaryngol Head Neck Surg. (1989) 100:6–16. 10.1177/019459988910000102 2493618

[19] Sumi T Watanabe I Tsunoda A Nishio A Komatsuzaki A Kitamura K. Longitudinal study of 29 patients with Meniere's disease with follow-up of 10 years or more. Acta Otolaryngol. (2012) 132:10–5. 10.3109/00016489.2011.627570 22054051

[20] Tokumasu K Nagoya M Watanabe Y Shojaku H Mizukoshi K. Epidemiological study of severe cases of Meniere's disease in Japan. Acta Otolaryngol Suppl. (1995) 520 (pt 2):415–8. 10.3109/00016489509125286 8749177

[21] Pyykkö I Nakashima T Yoshida Y Zou J Naganawa S. Meniere's disease: a reappraisal supported by a variable latency of symptoms and the MRI visualization of endolymphatic hydrops. BMJ Open. (2013) 3:e001555. 10.1136/bmjopen-2012-001555 23418296 PMC3586172

[22] Belinchon A Perez-Garrigues H Tenias JM. Evolution of symptoms in Ménière's disease. Audiol Neurootol. (2012) 17:126–32. 10.1159/000331945 21985844

[23] Zhang S Guo Z Tian E Liu D Wang J Kong W. Meniere disease subtyping: the direction of diagnosis and treatment in the future. Expert Rev Neurother. (2022) 22:115–27. 10.1080/14737175.2022.2030221 35057670

[24] Paparella M Griebie MS. Bilaterality of Meniere's disease. Acta Otolaryngol. (1984) 97:233–7. 10.3109/00016488409130984 6720298

[25] Greven AJ Oosterveld WJ. The contralateral ear in Meniere's disease: a survey of 292 patients. Arch Otolaryngol. (1975) 101:608–12. 10.1001/archotol.1975.00780390022006 1164225

[26] Kentala E Pyykkö I Auramo Y Juhola M. Database for vertigo. Otolaryngol Head Neck Surg. (1995) 112:383–90. 10.1016/S0194-59989570271-7 7870437

[27] Rasku J Pyykkö I Levo H Kentala E Manchaiah V. Disease profiling for computerized peer support of Ménière's disease. JMIR Rehabil Assist Technol. (2015) 2:e9. 10.2196/rehab.4109 28582248 PMC5454554

[28] Sintonen H. The 15D Instrument (2005). Available at: http://www.15d-instrument.net/service.cntum?pageId=110293 (accessed January 9, 2022).

[29] Pyykkö I Manchaiah V Zou J Levo H Kentala E. Vestibular syncope: a disorder associated with drop attacks in Ménière's disease. Auris Nasus Larynx. (2017) 44:690–5. 10.1016/j.anl.2017.03.023 28478076

[30] Pyykkö I Pyykkö N Zou J Manchaiah V. Vestibular drop attacks in Ménière's disease: a systematic review and meta-analysis of frequency, correlates, and consequences. J Int Adv Otol. (2022) 18:25–31. 10.3233/VES-201502 33935127

[31] EuroQol EQ-5D-3L. Available at: https://euroqol.org/eq-5d-instruments (accessed January 9, 2022).

[32] Lopez-Escamez JA Carey J Chung WH. Diagnostic criteria for Menière's disease. J Vestib Res. (2015) 25:1–7. 10.3233/VES-150549 25882471

[33] Phillips J Murdin L Grant K Shepstone L Sims E Rea P. Risk factors for the development of bilateral Ménière's disease. Otol Neurotol. (2023) 44:925–30. 10.1097/MAO.0000000000003984 37590874

[34] Clemmens C Ruckenstein M. Characteristics of patients with unilateral and bilateral Ménière's disease. Otol Neurotol. (2012) 33:1266–9. 10.1097/MAO.0b013e31826426b9 22858716

[35] Requena T Gazquez I Moreno A Batuecas A Aran I Soto-Varela A. Allelic variants in TLR10 gene may influence bilateral affectation and clinical course of Meniere's disease. Immunogenetics. (2013) 65:345–55. 10.1007/s00251-013-0683-z 23370977

[36] Kodama A Kitahara M Kitanishi T. Clinical findings in Menière's disease with bilateral fluctuant hearing loss. Acta Otolaryngol Suppl. (1995) 519:227–9. 10.3109/00016489509121911 7610875

[37] Thomas K Harrison MS. Long-term follow up of 610 cases of Menière's disease. Proc R Soc Med. (1971) 64:853–7. 10.1177/003591577106400823 5569327

[38] Eraslan Boz H Kirkim G Koçoglu K Çakir Çetin A Akkoyun M Güneri EA. Cognitive function in Meniere's disease. Psychol Health Med. (2023) 28:1076–86. 10.1080/13548506.2022.2144637 36369758

[39] Zheng Q Xu X Yu L Li J Tang L. Postoperative anxiety and its relationship with life quality in patients with Ménière's diseases [Chinese]. Zhong Nan Da Xue Xue Bao Yi Xue Ban. (2018) 43:662–7. 10.11817/j.issn.1672-7347.2018.06.014 30110010

[40] Lahmann C Henningsen P Brandt T Strupp M Jahn K Dieterich M. Psychiatric comorbidity and psychosocial impairment among patients with vertigo and dizziness. J Neurol Neurosurg Psychiatry. (2015) 86:302–8. 10.1136/jnnp-2014-307601 24963122

[41] Cha YH Baloh RW Cho C Magnusson M Song JJ Strupp M. Mal de Débarquement syndrome: diagnostic criteria. Consensus document of the Classification Committee of the Bárány Society. J Vestib Res. (2020) 30:285–93. 10.3233/VES-200714 32986636 PMC9249277

[42] Staab JP Eckhardt-Henn A Horii A Jacob R Strupp M Brandt T. Diagnostic criteria for persistent postural-perceptual dizziness (PPPD): consensus document of the committee for the Classification of Vestibular Disorders of the Bárány Society. J Vestib Res. (2017) 27:191–208. 10.3233/VES-170622 29036855 PMC9249299

[43] Pyykkö I Zou J Manchaiah V. Constant dizziness versus episodic vertigo in Ménière's disease: health-related quality of life, cognitive dissonance, and postural problems. J Int Adv Otol. (2024) XX:1–9. 10.5152/iao.2024.231113 39390907 PMC11562517

[44] Basura GJ Adams ME Monfared A Schwartz SR Antonelli PJ Burkard R. Clinical practice guideline: Ménière's disease. Otolaryngol Head Neck Surg. (2020) 162(2_suppl):S1–55. 10.1177/0194599820909438 32267799

[45] Edelman S Mahoney AE Cremer PD. Cognitive behavior therapy for chronic subjective dizziness: a randomized, controlled trial. Am J Otolaryngol. (2012) 33:395–401. 10.1016/j.amjoto.2011.10.009 22104568

[46] Holmberg J Karlberg M Harlacher U Magnusson M. One-year follow-up of cognitive behavioral therapy for phobic postural vertigo. J Neurol. (2007) 254:1189–92. 10.1007/s00415-007-0499-6 17676355

[47] Mahoney AE Edelman S Cremer PD. Cognitive behavior therapy for chronic subjective dizziness: longer-term gains and predictors of disability. Am J Otolaryngol. (2013) 34:115–20. 10.1016/j.amjoto.2012.09.013 23177378

[48] Popkirov S Staab JP Stone J. Persistent postural-perceptual dizziness (PPPD): a common, characteristic and treatable cause of chronic dizziness. Pract Neurol. (2018) 18:5–13. 10.1136/practneurol-2017-001809 29208729

[49] Dai M Cohen B Smouha E Cho C. Readaptation of the vestibulo-ocular reflex relieves the Mal De Debarquement syndrome. Front Neurol. (2014) 5:124. 10.3389/fneur.2014.00124 25076935 PMC4097942

[50] Micarelli A Viziano A Micarelli B Augimeri I Alessandrini M. Vestibular rehabilitation in older adults with and without mild cognitive impairment: effects of virtual reality using a head-mounted display. Arch Gerontol Geriatr. (2019) 83:246–56. 10.1016/j.archger.2019.05.008 31102927

[51] Kutlubaev MA Xu Y Manchaiah V Zou J Pyykkö I. Vestibular drop attacks in Ménière's disease: a systematic review and meta-analysis of frequency, correlates and consequences. J Vestib Res. (2021). 10.3233/VES-201514 33935127

[52] Baloh RW Jacobson K Winder T. Drop attacks with Menière's syndrome. Ann Neurol. (1990) 28:384–7. 10.1002/ana.410280314 2241120

[53] Stahle J Klockhoff I. Diagnostic procedures, differential diagnosis, and general conclusion. In:Pfaltz C, editor. Controversial Aspects of Meniere's Disease. Stuttgart: Thieme Verlag (1986). p. 71–86.

[54] Kwong E Pimlott N. Assessment of dizziness among older patients at a family practice clinic: a chart audit study. BMC Fam Pract. (2005) 6:1. 10.1186/1471-2296-6-2 15636636 PMC546211

[55] Newman-Toker DE Cannon LM Stofferahn ME Rothman RE Hsieh YH Zee DS. Imprecision in patient reports of dizziness symptom quality: a cross-sectional study conducted in an acute care setting. Mayo Clin Proc. (2007) 82:1329–40. 10.4065/82.11.1329 17976352

