# Supplementary material for: Changes in symptom pattern in Meniere's disease by duration: the need for comprehensive management
Source: Front Neurol. 2024 Nov 8;15:1496384. doi: 10.3389/fneur.2024.1496384 (PMC11581947; doi:10.3389/fneur.2024.1496384)
Supplement: Supplementary file 1 [file Data_Sheet_1.pdf]

Otoneurological Survey

All your answers are confidential and has the same protection as the case record has. It is important for research that you try to answer to all questions with care.

Personal Data

1. **Social security number:** ____________________

2. **Name:** ____________________

3. **Address:** ____________________
____

4. **Phone number:** ____________________

5. **Sex:**

Female	Male
☐	☐

Please mark the answer that best describes your health.

Answer to the questions concerning symptoms in a way that best describe the usual occurrence of them. If you don't have these particural symptoms nowadays, please answer the questions based on the situation when you previously had the symptoms.

6. **What symptoms do you have?** *(choose one or more options)*

vertigo	moving difficulties	hearing loss	tinnitus	headache
☐	☐	☐	☐	☐

Beginning of the Symptoms

7. **If you have or have previously had vertigo, hearing loss or tinnitus, with which symptoms did your disease start?** *(choose one or more options)*

vertigo	hearing loss	tinnitus	pressure feeling in the ear	moving difficulties
☐	☐	☐	☐	☐

8. **How old were you when the symptoms began?** ____________________

If you don't have vertigo, please move on to question 21.

9. If you have or have previously had vertigo and hearing loss, was there time difference between the attack of vertigo and hearing loss? *(choose only one option)*

1 = started at the same time	2 = less than year	3 = 1 - 4 years	4 = 5 - 10 years	5 = more than 10 years
☐	☐	☐	☐	☐

Vertigo

With vertigo episodes we mean temporary feeling of vertigo with few or no symptoms between the attacks.
With constant vertigo is meant continuous moving difficulties or ongoing feeling of vertigo.

10. Do you have these symptoms? *(choose one or several options)*

feeling of rotation	feeling of floating	tendency to fall	instability when moving	blackouts
☐	☐	☐	☐	☐

11. When did the first vertigo symptoms occur? *(choose one option)*

1 = less than month ago	2 = less than year ago	3 = 1 - 4 years ago	4 = 5 - 10 years ago	5 = more than 10 years ago
☐	☐	☐	☐	☐

12. What kind of vertigo do you have?

1 = constant	2 = episodes	3 = both
☐	☐	☐

If you have constant vertigo, please move on to the question 18.

13. How often do stronger episodes of vertigo occur?

1 = less than once a year	2 = less than once a month	3 = monthly	4 = weekly	5 = daily
☐	☐	☐	☐	☐

14. If you have vertigo attacks, how long do the stronger vertigo attacks last?

1 = less than 1 minute	2 = 1 - 10 minutes	3 = 20 minutes - 4 hours	4 = 4 hours - 24 hours	5 = more than day
☐	☐	☐	☐	☐

15. How severe are the vertigo attacks usually?

1 = mild (doesn't affect chores at all)	2 = weak (affects but can continue working normally)	3 = moderate (have to stop working)	4 = strong (must rest)	5 = very strong (difficulties despite rest)
☐	☐	☐	☐	☐

16. Do your vertigo attacks include nausea and/or vomiting?

0 = no	1 = weak	2 = moderate	3 = strong	4 = very strong vomiting
☐	☐	☐	☐	☐

17. Do you have sudden and strong drop attacks or falls lasting a second or two?

0 = no	1 = rarely	2 = less than once a week	3 = weekly	4 = daily
☐	☐	☐	☐	☐

18. Do changes in position induce vertigo?

0 = no	1 = weakly	2 = moderately	3 = strongly	4 = very strongly (falls)
☐	☐	☐	☐	☐

19. Do change in pressure (eg. flying, diving, blowing or sneezing) induce vertigo or balance difficulties?

0 = no	1 = weakly	2 = moderately	3 = strongly	4 = very strongly (falls)
☐	☐	☐	☐	☐

20. Does physical strain (eg. weight lifting) induce vertigo or balance difficulties?

0 = no	1 = weakly	2 = moderately	3 = strongly	4 = very strongly (falls)
☐	☐	☐	☐	☐

Mobility

21. Do you have balance or moving difficulties between attacks of vertigo?

0 = no	1 = rarely	2 =less than once a week	3 = weekly	4 = constantly
☐	☐	☐	☐	☐

22. If you have constant unsteadiness between attacks of vertigo, how severe is this?

0 = no handicap	1 = weak	2= moderate	3 = severe	4 = very severe (falls)
☐	☐	☐	☐	☐

23. Moving

0 = I can move normally	1 = I can move normally with little difficulty	2 = I can move with marked difficulty	3 = I can move only a little	4 = I am unable to move
☐	☐	☐	☐	☐

24. Standing up from the chair

0 = normally without using your hands	1 = with occasional help for your hands	2 = with your hands	3 = with help from others	4 = I can't get up
☐	☐	☐	☐	☐

Hearing Loss

25. Has your hearing deteriorated?

0 = no	1 = in right ear	2 = in left ear	3 = in both ears
☐	☐	☐	☐

If you don't have hearing loss, please move on to question 29.

26. If you feel that your hearing has deteriorated, how much time has passed from the beginning of your hearing loss?

1 = less than month ☐ 2 = less than year ☐ 3 = 1 - 4 years ☐ 4 = 5 - 10 years ☐ 5 = more than 10 years ☐

27. Does your hearing fluctuate during the vertigo attacks?

0 = no ☐ 1 = yes ☐

28. How did your hearing loss start?

1 = suddenly (in few days) ☐ 2 = over a few months ☐ 3 = over several years ☐

Tinnitus and Hyperacusis

With tinnitus is meant different sounds (eg. ringing, hum, feeling of pulse etc.) occuring in the ear or head, ie. tinnitus.
Hyperacusis refers to discomfort in the ear caused by strong voices or sensing voices very loud.

29. Which ear has the noise?

0 = no tinnitus ☐ 1 = right ear ☐ 2 = left ear ☐ 3 = bilateral ☐ 4 = noise is in whole head ☐

If you don't have tinnitus, please move on to question 33.

30. When did your tinnitus first occur?

1 = less than month ago ☐ 2 = less than a year ago ☐ 3 = 1 - 4 years ago ☐ 4 = 5 - 10 years ago ☐ 5 = more than 10 years ago ☐

31. How much handicap does tinnitus cause in your life?

0 = no handicap (doesn't affect chores) ☐ 1 = slight handicap (no real effect on normal life) ☐ 2 = moderate handicap (some disturbance on normal life) ☐ 3 = severe handicap (affects concentration and/or sleep) ☐ 4 = very severe handicap (completely disrupts my life) ☐

32. What type of tinnitus do you have?

1 = hum ☐ 2 = ring ☐ 3 = pulse ☐ 4 = buzz, hiss, stir ☐ 5 = other, several ☐

33. Do strong voices hurt you (hyperacusis)?

0 = no ☐ 1 = in the right ear ☐ 2 = in the left ear ☐ 3 = in both ears ☐

34. Handicap of hyperacusis

0 = no handicap	1 = mild	2 = moderate	3 = severe	4 = very severe
☐	☐	☐	☐	☐

35. Do you have a pressure feeling in the ear?

0 = no	1 = in the right ear	2 = in the left ear	3 = in both ears
☐	☐	☐	☐

Other Symptoms

36. Do you have other symptoms? *(choose one or several options)*

no other symptoms	feeling of faintness	feeling of drunkenness	blurring of eyes, growing black	feeling of unreality
☐	☐	☐	☐	☐

37. Handicap of other symptoms *(choose one alternative)*

0 = no handicap	1 = mild	2 = moderate	3 = severe	4 = very severe
☐	☐	☐	☐	☐

38. Anxiety or nervousness (Does vertigo, hearing loss or tinnitus cause these symptoms?)

0 = no	1 = slightly	2 = moderately	3 = severely	4 = very strongly
☐	☐	☐	☐	☐

39. Energetic

0 = I feel healthy	1 = I'm a little exhausted	2 = I feel fairly moderately tired	3 = I feel exhausted	4 = I feel totally exhausted
☐	☐	☐	☐	☐

Headache

If you have episodes of vertigo, please answer to next questions based on the headache outside the spells.

40. How long does headache last?

0 = no headache	1 = less than 2 hours	2 = 2 hours - 24 hours	3 = constant headache
☐	☐	☐	☐

If you don't have headaches outside attacks, please move on to question 43.

41. How often does the headache occur?

1 = less than once a year	2 =less than once a month	3 = monthly	4 = weekly	5 = daily
☐	☐	☐	☐	☐

42. Do you have headaches during the vertigo attacks?

0 = no	1 = rarely	2 = moderately	3 = frequently	4 = very frequently
☐	☐	☐	☐	☐

Neurological Symptoms

Do you have or suffer from next symptoms?

	0 = No	1 = Yes
a) Tend to faint (unconsciousness)	☐	☐
b) Blurring of your vision or double vision during attacks of vertigo	☐	☐
c) Weakness of your voice, stuttering or blurring of your speech (dysarthria) during attacks of vertigo	☐	☐
d) Difficulties in swallowing (cranial nerve palsy)	☐	☐
e) Fingling in your face or disturbance of sensitivity	☐	☐
f) Migraine which has been diagnosed by a physician	☐	☐

Alcohol

44. How many restaurant-sized measures of alcohol do you consume in a week?

0 = I don't use alcohol	1 =less than 4 units	2 = 5 - 9 units	3 = 10 - 20 units	4 = more than 20 units
☐	☐	☐	☐	☐

Oto- and Vestibulotoxic Drugs

Do you use or have had drugs for these?

	0 = No	1 = Yes
a) Heart medicines (diuretics)	☐	☐
b) Antibiotics by injection (apart from Penicillin)	☐	☐

46. Do you use strong pain killers eg. Burana?

0 = no	1 = occasionally	2 = weekly	3 = daily
☐	☐	☐	☐

Do you use or have had drugs for these?

	0 = No	1 = Yes
c) Treatment for malignant tumours/cancers	☐	☐
d) Tablets for depression	☐	☐
e) Other psychotopic drugs	☐	☐
f) Sleeping pills	☐	☐

Possible Damages of Internal Ear

Have you had any...

	0 = No	1 = Yes
a) Direct injury to the head or neck which was associated with the beginning of vertigo symptoms? (symptoms occured in 6 moths of the event)	☐	☐
b) Concussion with unconsciousness lasting less than 2 hours<br>in which year? ______	☐	☐
c) Brain injury with unconsciousness lasting more than 2 hours<br>in which year? ______	☐	☐
d) Whiplash injury<br>in which year? ______	☐	☐
e) An ear infection or prolonged (over three months) ear discharge / running ear caused by inflammation	☐	☐
f) Direct injury of the ear, acute noise injury, bleeding from the ear which would have caused hearing loss or tinnitus<br>in which year? ______	☐	☐
g) Been exposed to loud noise at work (noise level exceeding 85 dB) for more than 5 years	☐	☐

Ear Operations

49. Have you had a ear operation?

0 = no ☐ 1 = I don't know ☐ 2 = yes ☐

If you haven't had a ear operation, please move on to question 67.

50. Which ear was operated on?

1 = right ear ☐ 2 = left ear ☐ 3 = both ears ☐ 4 = I don't know ☐

If you know what has been operated on, please answer to following questions. If you don't know, please move on to question 67.

51. Have you had ear surgery because of vertigo?

0 = no .... ☐

1 = yes, ... ☐

what? ______

in which year? ______

Other Diseases

Do you have these diseases?

	0 = No	1 = Yes
a) Any symptoms of coronary heart disease	☐	☐
b) Hypertension/ high blood pressure	☐	☐
c) Arteriosclerosis	☐	☐
d) Any symptoms of brainstem ischemia	☐	☐
e) Kidney insufficiency/renal failure	☐	☐
f) Diabetes	☐	☐
g) Thyroid gland over- or underproduction	☐	☐
h) Have you suffered from meningitis or sequelae of mumps?	☐	☐

what? ______________________

in which year? ______________________

Family History

53. Does/did your father or mother have vertigo or hearing loss before the age of 65?

0 = no ☐ 1 = I don't know ☐ 2 = yes ☐

54. Do any of your siblings have vertigo or hearing loss before the age of 65?

0 = no ☐ 1 = I don't know ☐ 2 = yes ☐

55. Do your children have hearing loss?

0 = no ☐ 1 = I don't know ☐ 2 = yes ☐

56. If yes for something above, do you know the reason for the vertigo or hearing loss in your family?

1 = I don't know .... ☐

2 = yes, ... ☐

what? ______________________

57. How many siblings do you have?

0 = none ☐ 1 = one ☐ 2 = two ☐ 3 = three ☐ 4 = more than three ☐

ENRICHED QUALITY OF LIFE QUESTIONNAIRE (EQ-5D)

By placing a tick in one box in each group below, please indicate which statements best describe your own health state today.

Mobility

I have no problems in walking about ☐

I have some problems in walking about ☐

I am confined to bed ☐

Self-Care

I have no problems with self-care ☐

I have some problems washing and dressing myself ☐

I am unable to wash or dress myself ☐

Usual activities *(e.g.work, study, housework, family or leisure activities)*

I have no problems with performing my usual activities ☐

I have some problems with performing my usual activities ☐

I am unable to perform usual activities ☐

Pain/Distress

I have no pain or discomfort ☐

I have moderate pain or discomfort ☐

I have extreme pain or discomfort ☐

Anxiety/Depression

I am not anxious or depressed ☐

I am moderately anxious or depressed ☐

I am extremely anxious or depressed ☐
