# Supplementary material for: Changes in symptom pattern in Meniere's disease by duration: the need for comprehensive management
Source: Front Neurol. 2024 Nov 8;15:1496384. doi: 10.3389/fneur.2024.1496384 (PMC11581947; doi:10.3389/fneur.2024.1496384)
Supplement: Supplementary file 2 [file Data_Sheet_2.pdf]

Appendix Questionary 2

It is known that no two individuals with Meniere's disease have the exact same symptoms and problems. The variability of symptoms is significant and influenced by many factors, with different impacts. Factors modifying the course of the disease include family history of Meniere's disease, migraines, thyroid diseases, and rheumatic inflammations. Other modifying factors include head trauma, noise exposure, kidney dysfunction, high blood pressure, and cerebral circulation disorders, which currently are believed to increase balance issues. Diabetes seems to affect the quality of life rather than the actual symptoms of the disease. We aim to verify this through the questionnaire.

Additionally, the impact of medication on Meniere's symptoms varies between individuals, likely due to background factors. Some medications are found to be helpful, while others are not. Our research aims to develop more personalized treatments to find the best possible care for each individual with Meniere's disease.

The purpose of this new questionnaire is to investigate the effects of various factors on the disease's course and assess the impact of medication on different Meniere's symptoms. We kindly ask you to take time to complete the questionnaire, as the responses will help us develop individualized treatment and better understand the occurrence of different symptoms. The survey is anonymous.

By simply being a member of the Meniere Association, participating in local Meniere events or the Meniere Academy, using the MeniTuki program, or being in the Meniere's disease Facebook group, you are contributing to the collective work to support those with Meniere's disease. Active participation helps ensure continued support for Meniere activities.

The survey takes approximately 30 minutes to complete.

GENDER

- Female
- Male
- Other
- Prefer not to say

- Select one or more options.
  Digital participation means that you have taken part in Meniere activities online.

YEAR OF BIRTH

Answer…

HOW LONG HAVE YOU HAD MENIERE'S DISEASE?

Answer in numbers, e.g., if you have had it for 12 years, write: 12

WHEN WAS YOUR LAST MENIERE EPISODE? DATE OR ESTIMATE (e.g., '14.1.2021'):

IS THERE A FAMILY HISTORY OF MENIERE'S DISEASE BESIDES YOURSELF?

Family includes: parents, ancestors, siblings, children.

- No
- Yes. How many of your relatives have it?

WHAT ARE THE MAIN ISSUES LIMITING YOUR WORK OR FUNCTIONAL ABILITY?

Functional ability refers to everyday activities such as going to the store, public offices, or hobbies.
Mark the issues that limit your ability to function.

Rank the issues you marked by writing 1 for the most limiting problem, 2 for the next most limiting, etc.

- None
- Hearing impairment
- Dizziness
- Balance difficulties
- Tumarkin's attacks
- Tinnitus
- Pressure sensation
- Sound sensitivity
- Difficulty looking at computer or TV screens
- Other, what? Write your answer and rank it

HOW WOULD YOU RATE YOUR QUALITY OF LIFE ON A SCALE FROM 0 TO 100?

100 represents the best possible life and 0 represents the worst.

HAVE YOU BEEN DIAGNOSED WITH MIGRAINE BY A DOCTOR?

- No
- Yes

DO YOU HAVE HEADACHES?

- No
- Occasionally
- Frequently

DO YOU EXPERIENCE THE FOLLOWING VISUAL SYMPTOMS?

Mark those you have experienced:

- Zigzag patterns
- Moving black spots
- Blind spots or visual field defects
- None of the above

HAVE YOU EXPERIENCED THE FOLLOWING SYMPTOMS IN THE LAST TWO YEARS?

Mark those you have experienced:

- Spinning sensation
- Rocking sensation
- Tendency to fall
- Uncertainty while moving
- Loss of consciousness
- None of the above

WHAT KIND OF DIZZINESS HAVE YOU EXPERIENCED IN THE LAST TWO YEARS?

- None
- Episodic
- Continuous
- Both continuous and episodic

HAVE YOU EXPERIENCED ROTATIONAL DIZZINESS IN THE LAST TWO YEARS?

- None
- Less than once a year
- Less than once a month
- Monthly
- Weekly
- Daily

HOW LONG DO YOUR SEVERE DIZZINESS EPISODES TYPICALLY LAST?

- None
- Less than 1 minute
- 1 minute - 20 minutes
- 20 minutes - 4 hours
- 4 hours - 24 hours
- More than a day

HOW SEVERE ARE YOUR DIZZINESS EPISODES USUALLY?

- None
- Very mild: does not affect daily tasks or work at all
- Mild: affects but can continue daily tasks or work normally
- Moderate: must stop tasks or work
- Severe: must rest
- Very severe: difficulties even despite rest

HAVE YOU EXPERIENCED SUDDEN, SEVERE DIZZINESS EPISODES LASTING A FEW SECONDS WITHOUT HEAD MOVEMENT IN THE LAST TWO YEARS?

Sudden dizziness means that, even while at rest, you stumble suddenly and severely. This is also referred to as Tumarkin's attacks.

- None
- Occasionally
- Less than once a month
- Monthly
- Weekly

- Daily

HAVE SUDDEN DIZZINESS EPISODES CAUSED FALLS IN THE LAST TWO YEARS?

- No sudden dizziness episodes
- Have caused mild stumbling
- Would have fallen without support
- I have fallen

HAVE SUDDEN DIZZINESS EPISODES CAUSED YOU TO LOSE CONSCIOUSNESS IN THE LAST TWO YEARS?

- I have not had sudden dizziness episodes
- I have never lost consciousness
- I believe I have lost consciousness
- I have lost consciousness, confirmed by another person

HAVE YOU EXPERIENCED BALANCE OR MOVEMENT DIFFICULTIES OUTSIDE OF DIZZINESS EPISODES IN THE LAST TWO YEARS?

- No
- Yes

DOES IT FEEL LIKE THE GROUND IS SWAYING UNDER YOU WHILE STANDING OR WALKING?

- Sways slowly, about 1 sway every 3-5 seconds
- Sways quickly, about 1 sway per second
- No swaying, but my balance difficulties consist of stumbling
- No swaying and no stumbling

WHAT IS THE LIMITATION CAUSED BY YOUR BALANCE DIFFICULTIES?

- No balance difficulties
- Very mild: does not affect tasks

- Mild: affects, but I can continue moving normally
- Moderate: I must stop moving
- Severe: I must rest
- Very severe: difficulties despite rest

QUESTIONS RELATED TO MOOD AND ENERGY: DEPRESSION

1. I do not feel sad, down, or depressed at all.
2. I feel slightly sad, down, or depressed.
3. I feel moderately sad, down, or depressed.
4. I feel very sad, down, or depressed.
5. I feel extremely sad, down, or depressed.

QUESTIONS RELATED TO MOOD AND ENERGY: ANXIETY

1. I do not feel anxious, tense, or nervous at all.
2. I feel slightly anxious, tense, or nervous.
3. I feel moderately anxious, tense, or nervous.
4. I feel very anxious, tense, or nervous.
5. I feel extremely anxious, tense, or nervous.

QUESTIONS RELATED TO MOOD AND ENERGY: ENERGY LEVEL

1. I feel healthy and full of vitality.
2. I feel slightly fatigued, tired, or powerless.
3. I feel moderately fatigued, tired, or powerless.
4. I feel very fatigued, tired, or powerless, almost "burned out."
5. I feel extremely fatigued, tired, or powerless, completely "burned out."

HAVE YOU HAD A HEAD OR NECK INJURY OR EAR INFECTION LINKED TO THE ONSET OF DIZZINESS SYMPTOMS?

Dizziness symptoms started within 6 months of the event.

- No
- Yes
- I don’t know

HAVE YOU EXPERIENCED A CONCUSSION OR HEAD INJURY THAT RESULTED IN LOSS OF CONSCIOUSNESS?

Loss of consciousness lasted less than 2 hours.

- No
- Yes. What year? Enter the year.
- I don’t know

HAVE YOU HAD AN INJURY THAT RESULTED IN LOSS OF CONSCIOUSNESS FOR 2 HOURS OR MORE?

- No
- Yes. What year? Enter the year.
- I don’t know

HAVE YOU HAD A NECK INJURY?

For example, in an accident.

- No
- Yes. What year? Enter the year.

HAVE YOU HAD LONG-TERM DISCHARGE FROM THE EARS CAUSED BY AN INFECTION?

Long-term discharge lasting over 3 months.

- No
- Yes

HAVE YOU HAD AN EAR INJURY, TRAUMA, OR EXPLOSION THAT CAUSED DIZZINESS SYMPTOMS?

- No
- Yes. What year? Enter the year.

HAVE YOU BEEN EXPOSED TO LOUD NOISE FOR OVER 5 YEARS AT WORK, IN HOBBIES, OR DURING LEISURE TIME?

Noise level over 85 dB.

- No
- Yes

DO YOU HAVE ANY OF THE FOLLOWING DISEASES?

- Rheumatism
- Bowel disease
- Allergies
- Other autoimmune diseases
- Hypertension
- Cerebral circulation disorders
- Kidney failure
- Diabetes
- Thyroid overactivity or underactivity, or thyroid surgery
- None of the above

IF YOU HAVE OTHER AUTOIMMUNE DISEASES, WHAT ARE THEY?

Please describe in more detail what other autoimmune diseases you have, in addition to those listed earlier.

Your answer...

IF YOUR THYROID HAS BEEN TREATED, PLEASE EXPLAIN THE REASON AND WHETHER YOU ARE STILL BEING TREATED WITH MEDICATION, INCLUDING THE DOSAGE.

Your answer...

HAVE YOU RECEIVED ANY TREATMENT FOR DIZZINESS/BALANCE PROBLEMS IN THE LAST 2 YEARS?

- No
- Yes

- Medication
- Physiotherapy
- Psychotherapy
- Surgery

WHAT OTHER TREATMENTS, BESIDES THE PREVIOUSLY MENTIONED, HAVE YOU RECEIVED FOR DIZZINESS/BALANCE PROBLEMS?

Your answer...

WHAT SYMPTOM-SPECIFIC MEDICATION HAVE YOU USED FOR DIZZINESS, NAUSEA, OR FEELING UNWELL? PLEASE PROVIDE MORE DETAILS.

Medication name, dosage (whether taken orally or by injection), total daily dose, and duration of treatment: (single dose, as needed, days, or months). Start by listing the most important medication and continue in order of importance. If you do not use medication for Meniere’s disease, write "None" in the first field.

1. Medication name, administration method, dose, duration of treatment
   ____
2. Medication name, administration method, dose, duration of treatment
   ____
3. Medication name, administration method, dose, duration of treatment
   ____
4. Medication name, administration method, dose, duration of treatment
   ____
5. Medication name, administration method, dose, duration of treatment
   ____
6. Medication name, administration method, dose, duration of treatment
   ____
7. Medication name, administration method, dose, duration of treatment
   ____
8. Medication name, administration method, dose, duration of treatment
   ____
9. Medication name, administration method, dose, duration of treatment
   ____
