# Supplementary material for: Changes in symptom pattern in Meniere's disease by duration: the need for comprehensive management
Source: Front Neurol. 2024 Nov 8;15:1496384. doi: 10.3389/fneur.2024.1496384 (PMC11581947; doi:10.3389/fneur.2024.1496384)
Supplement: Supplementary file 3 [file Data_Sheet_3.pdf]

PART 1. PERSONAL INFORMATION AND BACKGROUND QUESTIONS ABOUT SYMPTOMS

1.2 I am:

- Female
- Male

1.3 My age (in numbers):*______________________

1.4 I am:

- Working full-time
- Working part-time
- On partial disability pension
- On full disability pension
- Unemployed
- Student
- Retired
- On sick leave
- Other

1.5 Education level:

- Primary education
- Secondary education (high school)
- Secondary education (vocational training)
- Higher education (polytechnic, university)

1.6 I have had Meniere's disease for:

- Less than 1 year
- 1-5 years
- 6-10 years
- 11-20 years
- 21-30 years
- Over 30 years

1.7 My first Meniere's episode occurred in the year (answer with the year, e.g., '2014'): ___________________

1.8 The most recent episode was on (answer with date, e.g., '12.11.2019'), or estimate if you don't remember: ________________________________

1.9 There are other people in my family with Meniere's disease:

- No
- Yes

1.10 How many people in your family do you know have Meniere's disease symptoms (answer in numbers)? __________________

1.11 What is the main issue limiting your work or functional ability?
Functional ability refers to everyday activities like going to the store, public offices, or hobbies. Select one or more options.*

- None
- Hearing impairment
- Dizziness
- Balance difficulties
- Tumarkin's attacks
- Fatigue
- Tinnitus or feeling of pressure
- Sound sensitivity
- Difficulty looking at computer or TV screens

1.12 Other, what? _______________________

1.13 How would you rate your quality of life on a scale from 0 to 100, where 100 is a full life: ___________________

1.14 Migraine

- I do not have migraine
- I have migraine

1.15 Headache

- I do not have frequent headaches:
- I have frequent headaches

1.16 I experience in my field of vision:

- Zigzag patterns
- Black moving spots
- Loss of vision, blind spots
- None of the above

1.17 In the visual field other, what? ____

1.20 In the last 2 years, I have experienced the following symptoms:

- Spinning sensation
- Rocking sensation
- Tendency to fall
- Uncertainty when moving
- Loss of consciousness

PART 2. DIZZINESS ATTACKS

These questions refer to symptoms experienced in the past 2 years. If no dizziness has occurred in the past 2 years, skip to Section 3. TUMARKIN'S ATTACKS.

2.1 What kind of dizziness have you experienced:

- I haven’t had dizziness in the last two years
- Continuous
- Episodic
- Both

2.2 Rotational dizziness:

- No rotational dizziness at all
- Occurs less than once a year
- Occurs less than once a month
- Occurs monthly
- Occurs weekly
- Occurs daily

2.3 How long does a typical severe rotational dizziness episode last:

- No dizziness episodes at all
- Less than 1 minute
- 1 minute - 20 minutes
- 20 minutes - 4 hours
- 4 hours - 24 hours
- More than a day

2.4 How severe are your rotational dizziness episodes usually?

- No dizziness episodes at all
- Very mild (does not affect daily tasks/work at all)
- Mild (affects, but can continue tasks/work normally)
- Moderate (have to stop tasks/work)
- Severe (have to rest)
- Very severe (difficulties even after rest)

2.5 Do changes in position cause dizziness?

- No
- Slightly
- Moderately
- Strongly
- Very strongly (I fell)

2.6 Do you experience any warning symptoms before a dizziness episode?

- No

- Yes

2.7 If you have warning symptoms, what are they and how long before do they occur? What symptoms? ____

PART 3. TUMARKIN'S ATTACKS

These are sudden balance losses lasting a second. The severity varies from a slight stumble to falls. In rare cases, the person's consciousness weakens, and they may be momentarily unconscious.

These questions refer to symptoms experienced in the past 2 years. If no dizziness has occurred in the past 2 years, skip to Section 4. BALANCE DIFFICULTIES AND INSTABILITY WHEN MOVING.

3.1 Do you experience sudden Tumarkin's attacks?

- No
- Occasionally
- Less than once a month
- Monthly
- Weekly
- Daily

3.2 Tumarkin's attacks:

- I don’t have Tumarkin's attacks
- Have led to a mild stumble
- Would have fallen without support
- I have fallen

3.3 As a result of Tumarkin's attacks:

- I don’t have Tumarkin's attacks
- I have not been injured
- I have been injured

3.4 If I have been injured, how: ___________________________________

3.5 Consciousness and attacks. Select one or more options:

- I don’t have Tumarkin's attacks
- I have never lost consciousness in a Tumarkin's attack
- I have temporarily lost consciousness in a Tumarkin's attack
- Another person has confirmed my unconsciousness during a Tumarkin's attack

3.6 Tumarkin's attacks:

- I don’t have Tumarkin's attacks
- They do not limit my life
- They limit my work or functional ability
- They make social life difficult
- They cause fear or anxiety

PART 4. BALANCE DIFFICULTIES AND INSTABILITY WHEN MOVING

If you do not have balance difficulties or instability outside of dizziness attacks, skip to Section 5. ADDITIONAL COMMENTS AND EXPERIENCES.

4.1 Do you have balance difficulties or movement difficulties outside of dizziness attacks?

- No
- Occasionally
- Less than once a week
- Weekly
- Constantly

4.2 When standing or walking, it feels like the ground is sway8ing or rocking beneath me:

- Not swaying or rocking
- Slow, about 1 sway every 3-5 seconds
- Fast, about 1 sway per second
- My balance difficulties consist of stumbles

4.3 How do you stumble:

- I don’t stumble
- I stumble in all directions
- I mostly stumble in one direction

4.4 Effect of balance difficulties on vision:

- Everything in my field of vision stays still
- When I move my gaze, objects in my field of vision shift for a moment due to dizziness
- Objects and the environment seem to float or sway like in waves or wind
- I find it difficult to focus my gaze
- Judging distances becomes difficult

4.5 Do your balance difficulties improve when:

- Cycling
- Driving

- On a train
- On a ship
- On a plane
- No, movement only worsens symptoms

4.6 The limitation caused by my balance difficulties is:

- No balance difficulties
- Very mild (does not affect tasks at all)
- Mild (affects, but can continue moving normally)
- Moderate (have to stop moving)
- Severe (have to rest)
- Very severe (difficult even with rest)

PART 5. ADDITIONAL COMMENTS AND EXPERIENCES

6.1 Do you have any additional comments, experiences, or observations related to your condition, treatment, or the quality of your life? (Open-ended question): _____________
____
____
