# Supplementary material for: Changes in symptom pattern in Meniere's disease by duration: the need for comprehensive management
Source: Front Neurol. 2024 Nov 8;15:1496384. doi: 10.3389/fneur.2024.1496384 (PMC11581947; doi:10.3389/fneur.2024.1496384)
Supplement: Supplementary file 4 [file Table_1.docx]

***Appendix Table 1*** *Various drugs and therapies used by participants (n=365) in the study, including surgical and intratympanic (IT) therapies. The table also shows the effectiveness of these treatments in relation to achieving a vertigo-free period of more than 2 years. The effectiveness is analysed using the Kruskal-Wallis test for comparing multiple groups. ELS-surgery indicates endolymphatic sac surgery.*

| *Therapeutic approach* | *User percentage* | *No vertigo for 2 years* | *With vertigo attacks* | *Kruskal-Wallis test* |
| --- | --- | --- | --- | --- |
| Betahistin | 61.9*%* | 91 | 135 | p=0.686 |
| Diuretic | 20.5*%* | 31 | 44 | p=0.709 |
| Antiemetics | 40.3*%* | 46 | 101 | p=0.009 |
| IT-steroids | 2.2*%* | 3 | 5 | p=0.909 |
| IT-gentamicin | 2.7*%* | 7 | 3 | **p=0.045** |
| Antidepressants | 9.3*%* | 19 | 15 | **p=0-040** |
| ELS-surgery | 1.4*%* | 1 | 4 | p=0.371 |
| Psychotherapy | 4.4*%* | 7 | 9 | p=0.719 |
| Physiotherapy | 23.8*%* | 20 | 67 | **p<0.001** |
