# Supplementary material for: Changes in symptom pattern in Meniere's disease by duration: the need for comprehensive management
Source: Front Neurol. 2024 Nov 8;15:1496384. doi: 10.3389/fneur.2024.1496384 (PMC11581947; doi:10.3389/fneur.2024.1496384)
Supplement: Supplementary file 5 [file Table_2.docx]

Appendix figure legends.

*Figure 1. Latency between vertigo and hearing loss among the subjects.*

*Figure 2. Character of vertigo and dizziness among subjects.*

*Figure 3. Frequency of vertigo attacks in participants rated from no attacks to daily attacks in percent of the subjects.*

*Figure 4a. Character of vestibular drop attack. Only 10% fell to the ground. Another 10 % could prevent from falling by grabbing support. Figure 7b. Frequency of vestibular drop attacks in percent of the subjects.*

*Figure 5. Health related quality of life among patients with no vertigo, those with vertigo with or without constant dizziness. Means and SE are shown.*
