# Supplementary figures and images for: Changes in symptom pattern in Meniere's disease by duration: the need for comprehensive management
Source: Front Neurol. 2024 Nov 8;15:1496384. doi: 10.3389/fneur.2024.1496384 (PMC11581947; doi:10.3389/fneur.2024.1496384)

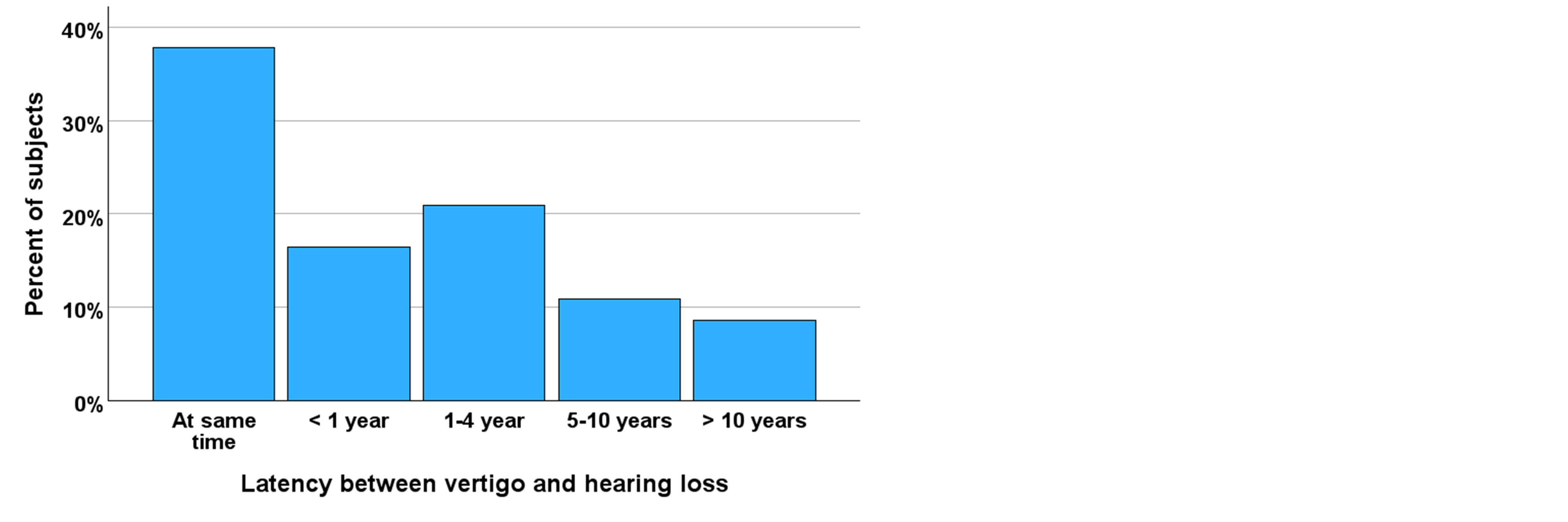

Supplement: Supplementary file 6 [file Image_1.tif]

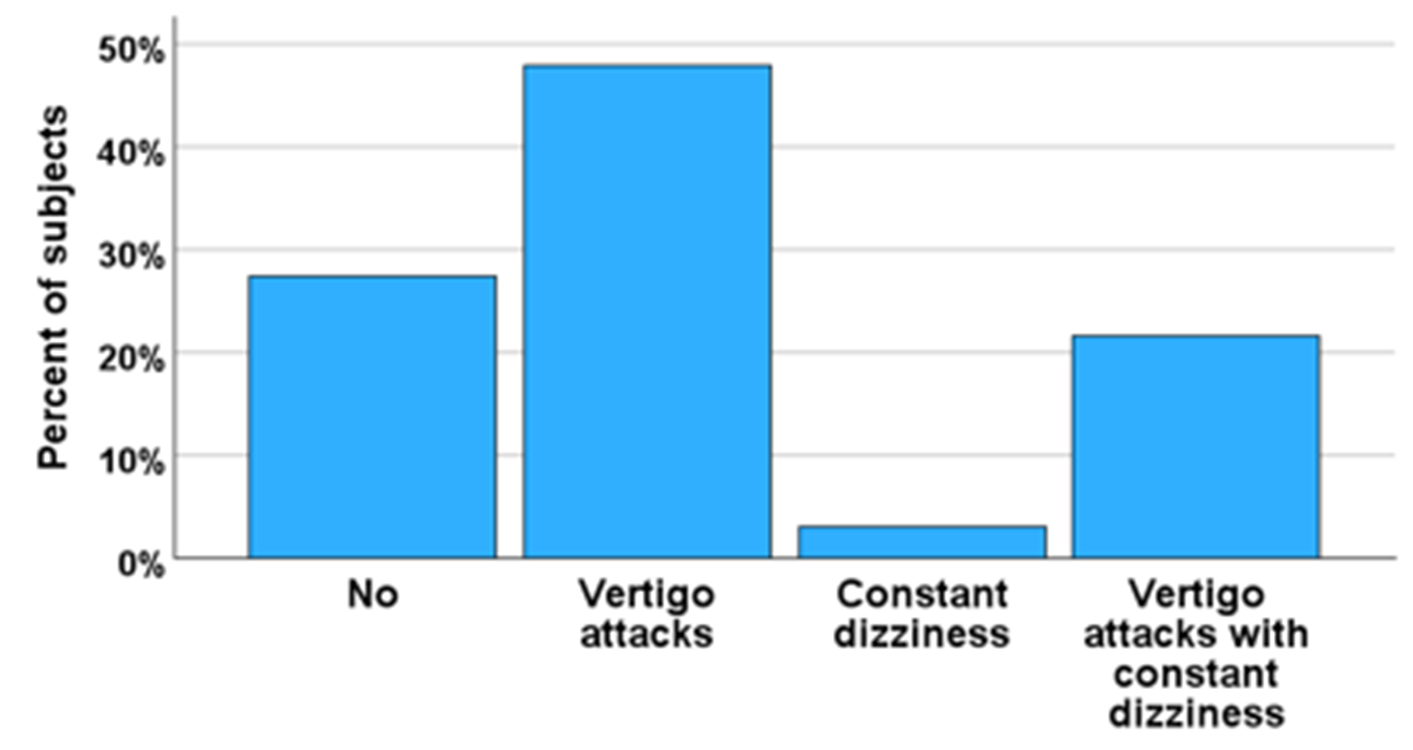

Supplement: Supplementary file 7 [file Image_2.tif]

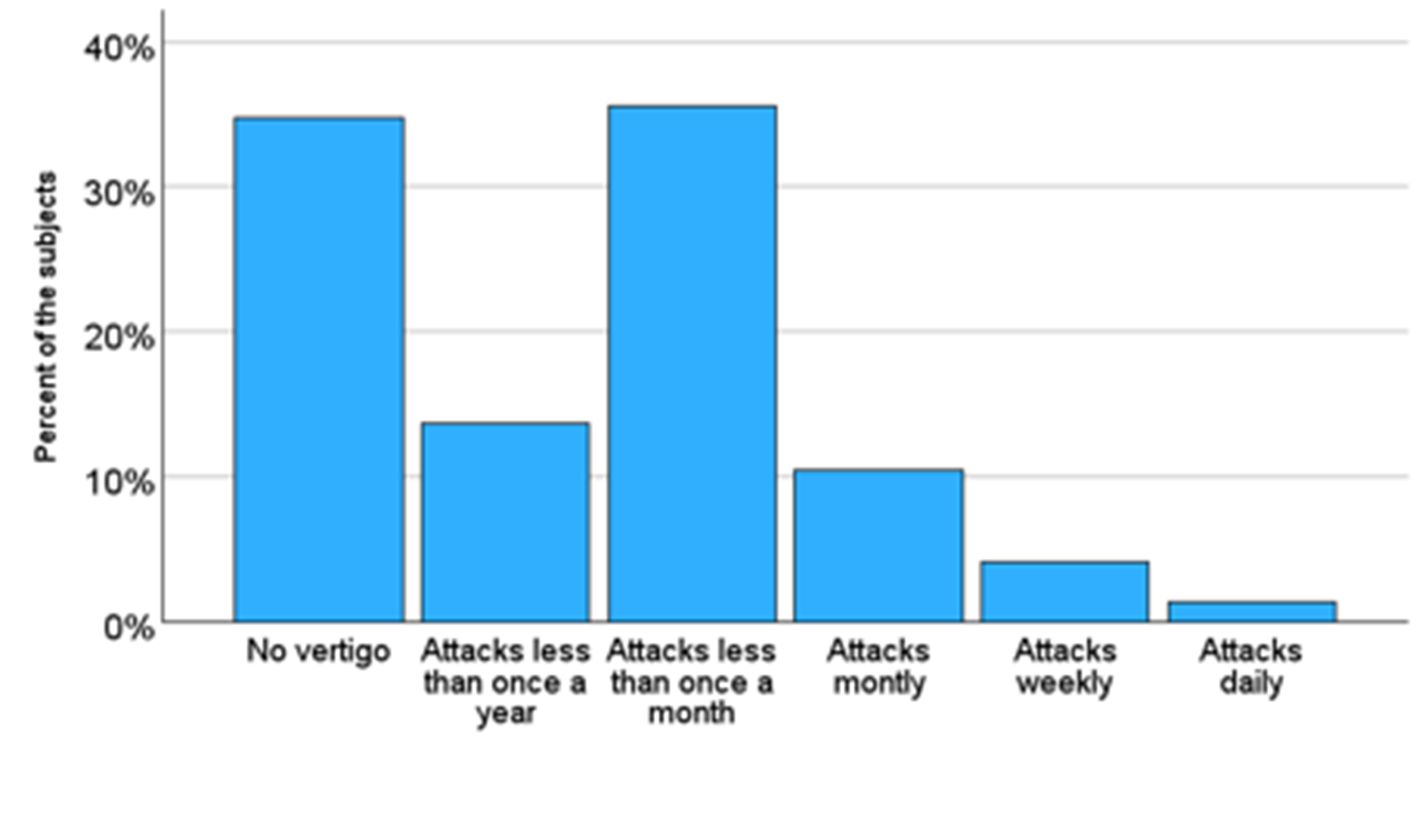

Supplement: Supplementary file 8 [file Image_3.tif]

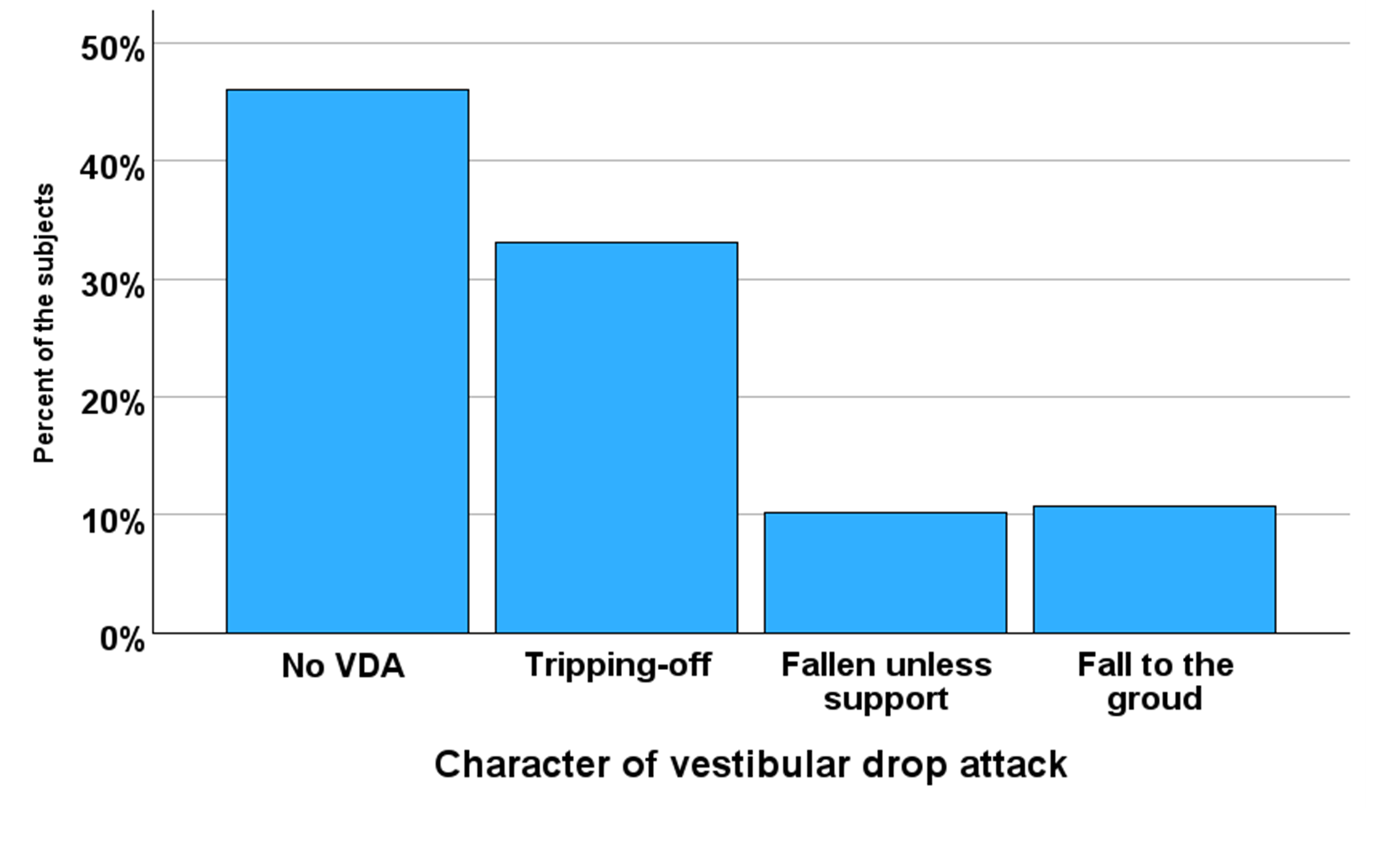

Supplement: Supplementary file 9 [file Image_4.tif]

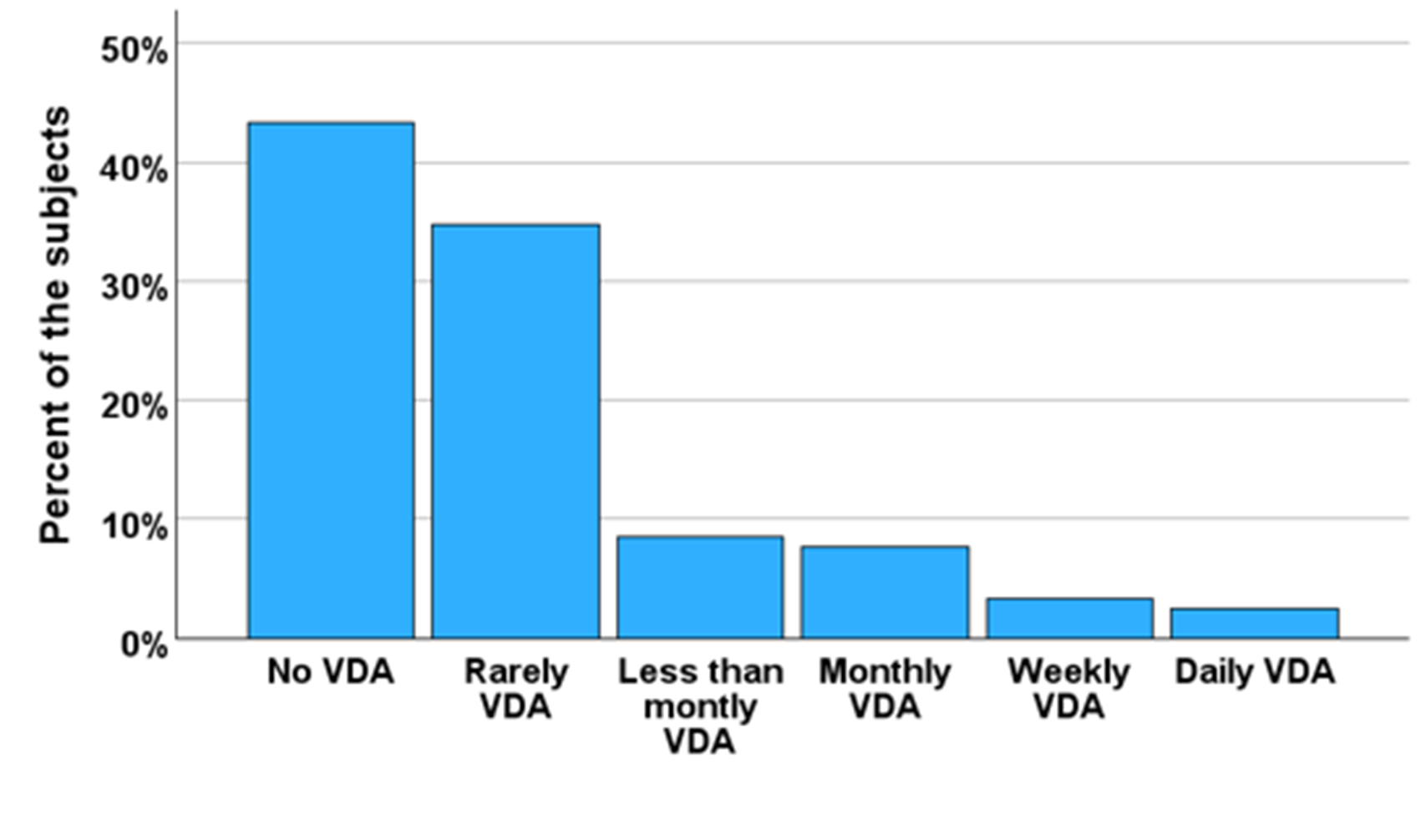

Supplement: Supplementary file 10 [file Image_5.tif]

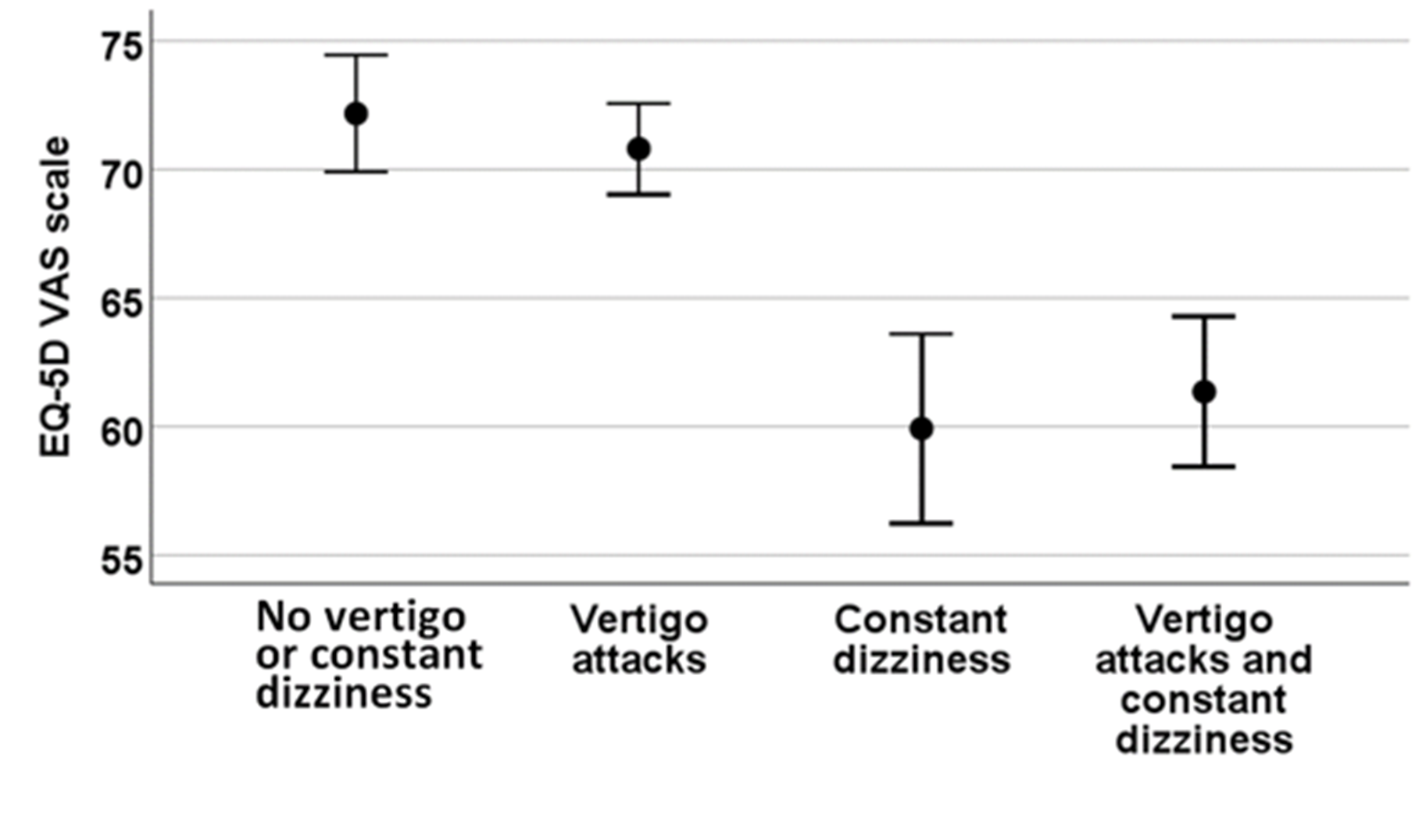

Supplement: Supplementary file 11 [file Image_6.tif]
